# Preclinical evaluation of local prolonged release of paclitaxel from gelatin microspheres for the prevention of recurrence of peritoneal carcinomatosis in advanced ovarian cancer

**DOI:** 10.1038/s41598-019-51419-y

**Published:** 2019-10-16

**Authors:** Kaat De Clercq, Feifan Xie, Olivier De Wever, Benedicte Descamps, Anne Hoorens, An Vermeulen, Wim Ceelen, Chris Vervaet

**Affiliations:** 1 Laboratory of Pharmaceutical Technology, Ghent University, Ottergemsesteenweg 460, 9000 Ghent, Belgium; 2 Laboratory for Medical Biochemistry and Clinical Analysis, Ghent University, Ottergemsesteenweg 460, 9000 Ghent, Belgium; 3 Laboratory of Experimental Cancer Research, Ghent University, Corneel Heymanslaan 10, 9000 Ghent, Belgium; 4 Infinity (IBiTech-MEDISIP), Department of Electronics and Information Systems, Ghent University, Corneel Heymanslaan 10, 9000 Ghent, Belgium; 5 Department of Pathology, Ghent University Hospital, Corneel Heymanslaan 10, 9000 Ghent, Belgium; 6 Department of Gastro-intestinal Surgery, Ghent University Hospital, Corneel Heymanslaan 10, 9000 Ghent, Belgium; 7 Cancer Research Institute Ghent (CRIG), Ghent, Belgium

**Keywords:** Drug development, Preclinical research, Translational research

## Abstract

Patients with advanced ovarian cancer develop recurrence despite initial treatment response to standard treatment of surgery and intravenous/intraperitoneal (IP) chemotherapy, partly due to a limited peritoneal exposure time of chemotherapeutics. Paclitaxel-loaded genipin-crosslinked gelatin microspheres (PTX-GP-MS) are evaluated for the treatment of microscopic peritoneal carcinomatosis and prevention of recurrent disease. The highest drug load (39.2 µg PTX/mg MS) was obtained by immersion of GP-MS in aqueous PTX nanosuspension (PTX_nano_-GP-MS) instead of ethanolic PTX solution (PTX_EtOH_-GP-MS). PTX release from PTX-GP-MS was prolonged. PTX_nano_-GP-MS displayed a more controlled release compared to a biphasic release from PTX_EtOH_-GP-MS. Anticancer efficacy of IP PTX-GP-MS (PTX_EtOH_-GP-MS, D = 7.5 mg PTX/kg; PTX_nano_-GP-MS D = 7.5 and 35 mg PTX/kg), IP nanoparticular albumin-bound PTX (D = 35 mg PTX/kg) and controls (0.9% NaCl, blank GP-MS) was evaluated in a microscopic peritoneal carcinomatosis xenograft mouse model. PTX_nano_-GP-MS showed superior anticancer efficacy with significant increased survival time, decreased peritoneal carcinomatosis index score and ascites incidence. However, prolonged PTX release over 14 days from PTX_nano_-GP-MS caused drug-related toxicity in 27% of high-dosed PTX_nano_-GP-MS-treated mice. Dose simulations for PTX_nano_-GP-MS demonstrated an optimal survival without drug-induced toxicity in a range of 7.5–15 mg PTX/kg. Low-dosed PTX_nano_-GP-MS can be a promising IP drug delivery system to prevent recurrent ovarian cancer.

## Introduction

The majority of ovarian cancer (OC) patients (>70%) is diagnosed at an advanced stage of the disease (International Federation of Gynecology and Obstetrics (FIGO) stage III or IV) with peritoneal metastases present since there are no specific symptoms and effective screening methods are lacking. The main symptoms are abdominal pain and swelling, gastrointestinal complaints and occasionally urinary symptoms or vaginal bleeding^1–6^. The stage of OC strongly determines survival; prognosis at FIGO stages III and IV is poor with a 5-year overall survival rate of 27 and 21%, respectively^1,7^.

Standard combination therapy of cytoreductive surgery (CRS) followed by six cycles of intravenous (IV) platinum- and taxane-based chemotherapy initially achieves complete remission in 80% of advanced OC patients. Unfortunately, most of the patients will relapse within 5 years because minimal residual disease persists in the peritoneal cavity despite a (nearly) complete cytoreduction. Adjuvant intraperitoneal (IP) chemotherapy has been added to the treatment modality of selected patients to improve treatment outcome since metastatic disease is mainly limited to the abdominal cavity. IP chemotherapy can be administered postoperatively via a catheter or as a (hyperthermic) intraoperative chemoperfusion ((H)IPEC) as a part of the surgical procedure^1,3,8,9^. The rationale of IP drug delivery is based on the existence of a peritoneal-plasma barrier. Peritoneal tumors can be exposed to a higher concentration of the cytotoxic agent with moderate systemic drug exposure, thereby reducing toxic side effects since the peritoneal clearance is much slower than the systemic clearance^8–11^. Paclitaxel (PTX) is theoretically an ideal chemotherapeutic agent for IP application as it has a peritoneal/plasma concentration ratio of >1000 and displays a significant first pass effect^9,12–14^.

Several large, prospective randomized phase III clinical trials showed that adjuvant IP chemotherapy significantly improved overall survival of patients with advanced ovarian cancer^2,15–17^. However, the risk of recurrent peritoneal disease remains high^18,19^. This can be partly attributed to a short residence time of the chemotherapeutics in the abdominal cavity and the single administration of HIPEC^18,20^. Up to now, there are no products approved by the US Food and Drug Administration (FDA) specifically for IP application. Consequently, current IP therapy is based on off-label use of IV chemotherapeutics. These conventional low molecular weight drugs are rapidly absorbed from the peritoneal cavity and lack tumor specificity^8,20,21^. Additionally, IP chemotherapy is only recommended for patients with optimally debulked peritoneal disease or microscopic disease because of the limited penetration depth of drugs directly into the tumor by free-surface diffusion^8,10,21^. A prolonged residence time of chemotherapeutics would avoid rapid clearance of the drug from the peritoneal cavity, thereby maintaining a local drug concentration gradient as the driving force for drug diffusion into the tumor, improving tumor penetration since drug diffusion is a slow process^10,20^.

Drug delivery systems designed for IP application have been investigated to overcome the challenges of IP therapy and further improve treatment of advanced ovarian cancer. Drug delivery strategies include microspheres, nanoparticles, liposomes, micelles, implants and injectable depots^10,13^. Nanoparticulate systems (nanoparticles, liposomes, micelles) seem interesting delivery systems for IP administration since they can be easily modified for targeted drug delivery and can incorporate hydrophobic chemotherapeutic agents. Two nanoparticulate formulations have been applied in phase I clinical trials for IP use, NanoTax^®^, a 600–700 nm rod shaped reservoir of PTX, and Abraxane^®^, nanoparticular albumin-bound PTX (nab-PTX) with a size of 130 nm. Both studies showed higher levels of PTX in the peritoneal cavity compared with IV administrated PTX^18^. But, due to their small size, they are rapidly absorbed from the abdominal cavity and their residence time is often insufficient to permit efficient tumor penetration^8,18,20^. Therefore, dispersion of a nanoparticulate system into a depot system might be an interesting approach to overcome these limitations. Microparticles, on the other hand, are retained in the peritoneal cavity because of their size (>1 µm). Drugs can be gradually released over time, thereby improving local bioavailability and reducing systemic toxicity. It should be noted that larger particles tend to induce inflammatory responses and peritoneal adhesions^8,20,22^. Paclimer^®^, biodegradable PTX microspheres based on a polyphosphoester polymer, was evaluated in a phase I clinical trial in recurrent ovarian cancer. A continuous IP PTX release over 8 weeks was demonstrated but the product was discontinued because of inflammatory responses due to the presence of residual polymer filaments^23^. Hydrogels can also form an IP sustained release depot. As homogenous distribution of the formulation throughout the peritoneal cavity is difficult to achieve using hydrogels due to viscosity issues, thermosensitive hydrogels were developed which are liquid at room temperature and form a non-flowing gel *in situ*^20,24,25^. Many biodegradable hydrogels loaded with a chemotherapeutic agent have been successfully developed and evaluation in peritoneal carcinomatosis models in animals showed superior antitumoral activity compared to the unentrapped cytostatic drug^26–29^. However, an injectable hydrogel formulation of PTX, Oncogel^®^, failed to show an impact on overall tumor response in a phase II b clinical trial following intratumoral administration despite good tolerance, favorable pharmacokinetics and preliminary anticancer activity^30,31^. Currently, there are no ongoing clinical trials investigating the potential of depot systems in the treatment of ovarian cancer.

Previously, genipin-crosslinked gelatin microspheres (GP-MS) were developed for IP application and were shown effective for the prevention of peritoneal adhesions, a common complication after CRS^32^. In the present study paclitaxel (PTX) is encapsulated in GP-MS, and its drug release behavior is studied. The effect of PTX-loaded GP-MS on the viability of ovarian cancer cell lines is also investigated. The efficacy of a prolonged IP PTX release from different types of PTX-loaded GP-MS (PTX-GP-MS) to eradicate microscopic peritoneal disease of ovarian origin and further prevent recurrence of peritoneal disease is evaluated in a human xenograft mouse model.

## Materials and Methods

### Incorporation of paclitaxel into genipin-crosslinked gelatin microspheres

Genipin-crosslinked gelatin microspheres with an average size of 50 µm were prepared as described in De Clercq *et al*., 2016.

GP-MS were loaded with PTX by immersion in an ethanolic PTX solution. 200 mg GP-MS with different degrees of crosslinking (7, 25, 40, 60 and 70%) were immersed in 2 ml of a 1, 2.5 or 5 mg/ml PTX (purity of >99%, LC laboratories, Woburn, MA, USA) ethanol/distilled water (75/25, *v/v*) (absolute ethanol, VWR chemicals, Fontenay-sous-Bois cedex, France) solution under slow magnetic stirring. After 3 hours, GP-MS were vacuum filtered and washed with absolute ethanol (VWR chemicals) to remove unentrapped PTX from the surface. PTX-loaded GP-MS were lyophilised for 24 hours at −50 °C and 1 mbar.

GP-MS were also loaded by immersion in an aqueous PTX-nanosuspension. Initially, PTX-nanocrystals were prepared using a wet milling technique^13^. Pluronic^®^ F-127/PTX (Pluronic^®^ F-127, Sigma-Aldrich, Bornem, Belgium) (PTX purity of >99%, LC laboratories) in a 1/4 ratio was transferred in a 20 ml vial containing 5 ml 0.9% sodium chloride solution (Sigma-Aldrich) and 30 g zirconium oxide beads (Netzsch zetabeads, Ghislenghien, Belgium) with a diameter of 0.5 mm as milling pearls. The vials were placed on a roller-mill and grinded at 150 rpm for 60 hours. The nanocrystals were lyophilised for 24 hours at −50 °C and 1 mbar. Particle size and polydispersity index (PI) of the PTX nanocrystals were determined by dynamic light scattering, using a Zetasizer 3000 (Malvern Instruments, Worcestershire, UK). GP-MS (50, 100 or 200 mg) were immersed for 3 hours in 2 ml aqueous PTX nanosuspension, diluted using 0.9% sodium chloride solution to a concentration of 1, 2.5 or 5 mg PTX/ml. PTX-loaded GP-MS were collected by vacuum filtration and washed using distilled water to remove unentrapped PTX from the surface. PTX_nano_-GP-MS were lyophilised for 24 hours at −50 °C and 1 mbar.

Loading efficiency of PTX in GP-MS was analysed by an in-house UPLC-MS/MS after degradation of the microspheres. To a test tube containing 10 mg blank GP-MS, 960 µl 2 N hydrogen chloride (purity of 37%, Sigma-Aldrich) was added and 40 µl of a PTX standard was spiked. PTX-GP-MS were weighed (10 mg) in a test tube and 1 ml 2 N hydrogen chloride (purity of 37%, Sigma-Aldrich) was added^33^. After incubating the samples for 2 hours at 60 °C, 4 ml ethanol (VWR chemicals) was added. Subsequently, the samples were vortexed and centrifuged at 3000 rpm for 5 minutes. The collected supernatant (500 µl) was transferred to a glass vial (Filter Service, Eupen, Belgium) with insert (Agilent Technologies, Diegem, Belgium), to which 500 µl deionized water (Synergy 185-millipore, Prod 18.2 MΩ.cm, Bedford, USA) and 50 µl IS, were added. After vortexing, the vials were placed in the autosampler thermostated at 5 °C. Aliquots of 1 µl were injected for analysis. After integration of the peaks, the calculated concentration of incorporated PTX, expressed as µg/ml, was converted to the amount of incorporated PTX (µg) per mass (mg) MS and the incorporation efficiency (IE) of PTX in MS was calculated using Eq. 1.

1 $${\rm{IE}}\,( \% )=\frac{{\rm{amount}}\,{\rm{of}}\,{\rm{PTX}}\,{\rm{per}}\,{\rm{mg}}\,{\rm{MS}}}{{\rm{maximum}}\,{\rm{amount}}\,{\rm{of}}\,{\rm{PTX}}\,{\rm{per}}\,{\rm{mg}}\,{\rm{MS}}}\times 100$$

### *In vitro* PTX release from paclitaxel-loaded GP-MS

*In vitro* release of PTX from PTX-loaded GP-MS was evaluated by a membrane-less method. 20 mg lyophilised PTX-loaded GP-MS was immersed in 10 ml release medium, phosphate buffered saline buffer (PBS) with a pH of 7.4 supplemented with 0.1% w/v polysorbate 80 (Tween 80, Fagron, Nazareth, Belgium) in closed test tubes. Drug release experiments were performed at 37 °C for 21 days under gentle shaking conditions. At specific time points, test tubes were centrifuged, and 1 ml of release medium was sampled. The withdrawn medium was replaced by 1 ml of fresh buffer solution. Before sampling, test tubes were centrifuged to avoid pipetting of GP-MS. Each experiment was performed in triplicate.

Concentrations of PTX and its stereoisomer 7-epi PTX in the release medium were determined by UPLC-MS/MS. After integration of the peaks, total cumulative concentration of released PTX (sum of PTX and 7-epi PTX), expressed as ng/ml, was calculated. Percentage PTX release (%) was calculated using Eq. 2.

2 $${\rm{PTX}}\,{\rm{release}}\,( \% )=\frac{{\rm{total}}\,{\rm{cumulative}}\,{\rm{concentration}}\,{\rm{of}}\,{\rm{released}}\,{\rm{PTX}}\,(\mathrm{ng}/\mathrm{ml})}{{\rm{maximum}}\,{\rm{released}}\,{\rm{PTX}}\,{\rm{concentration}}\,(\mathrm{ng}/\mathrm{ml})}\times 100$$

### Influence of PTX-loaded GP-MS on ovarian cancer cell viability

Human ovarian cancer (OC) cell lines SK-OV-3 and OVCAR-3 were purchased from American Type Culture Collection. SK-OV-3 Luc IP1 is isolated after *in vivo* selection of intraperitoneal (IP) injected SK-OV-3 Luc cells^34^. OVCAR-3 was cultured in RPMI 1640 medium (Life Technologies, ThermoFisher, Ghent, Belgium) supplemented with 10% fetal bovine serum (FBS, Sigma-Aldrich) and 2% penicillin/streptomycin (Life technologies). SK-OV-3 (Luc IP1) were cultured in Dulbecco’s Modified Eagle’s Medium (DMEM, Life Technologies), supplemented with 10% FBS (Sigma-Aldrich) and 2% penicillin/streptomycin (Life Technologies).

CellTiter Glo 2.0 Luminescent Assay was performed to determine cell viability based on quantification of adenosine triphosphate (ATP). OC cells were seeded as monolayers in opaque white 96-well plates (ThermoFisher). Seeding density depended on incubation time and is displayed in Table 1. Subsequently, after 24 hours cells were exposed to multiple concentrations of different types (7, 25, 40 and 60% degree of crosslinking) of PTX-ethanolic loaded GP-MS (PTX_EtOH_-GP-MS). Cells were also exposed to a concentration range of GP-MS loaded with PTX-nanosuspension (PTX_nano_-GP-MS) and to a PTX solution. PTX-loaded GP-MS were dispersed in an appropriate medium and diluted until a concentration of 1 to 1000 nM of PTX was achieved. 10 µl of test formulation was added to the cells. Untreated cells were used as a control. After 24, 72, 168 or 336 hours of incubation, 100 µl CellTiter Glo 2.0 Reagens (Promega, Leiden, The Netherlands) was added to each well. Luminescence was measured after 10 minutes using a Paradigm Detection Platform and analysed with Soft Max Pro 6.1 Software (BIO-RAD laboratories, Hemel Hempstead, United Kingdom). All experiments were performed in triplicate. The cytotoxic inhibitory concentration 50% (IC_50_)-values were calculated using nonlinear regression analysis (dose-response inhibition) in Graphpad Prism™ 6 (Graphpad software, La Jolla, CA, USA). Statistical analysis was performed using post-hoc Bonferroni test, a p-value < 0.05 was considered statistically significant.

**Table 1:** Seeding density of cells.

Incubation time (day)	Cells/well
1	30 000
2	25 000
4	20 000
7	10 000
14	5 000

### Efficacy of PTX-microspheres in a microscopic peritoneal carcinomatosis xenograft model

All animal experiments were approved by the Animal Ethics Committee of the Faculty of Medicine at Ghent University (ECD 17/83) and were performed according to Belgian and European legislature on animal welfare. Mice were kept in standard housing conditions with water and food *ad libitum* and a 12 h light/dark cycle. Mice were evaluated daily for pain or discomfort, and “The Guidelines for the welfare and use of animals in cancer research” were strictly followed for distention of the abdomen, general condition and other clinical signs as prescribed by The National Cancer Research Institute. All procedures were performed under general anesthesia (Sevorane^®^, Abbott, Belgium or Isoflo^®^, Ecuphar, Oostkamp, Belgium), and analgesia (ketoprofen, 5 mg/kg) was administered if necessary.

Six-week old female Balb/c Nu mice (BALB/cOlaHsd-Foxn1nu, Envigo, Horst, The Netherlands) were conditioned one week before the start of the study. A microscopic peritoneal carcinomatosis model was used to evaluate the effect of IP treatment on microscopic disease similar to the post-cytoreductive surgery state and the potential to prevent recurrent disease^35–37^. Mice were injected IP with 2 × 10^6^ SK-OV-3-Luc IP1 cells suspended in 1 ml 0.9% saline. Mice (10–12/group) were injected IP with PTX treatment; 50 mg of PTX-GP-MS suspended in 2 ml of 0.9% saline or nab-PTX (Abraxane^®^, Celgene Europe, Uxbridge, UK) 1 day after engraftment with tumor cells, referred to as day 0 of the study. Control animals (11/group) were IP injected with 2 ml of 0.9% saline or 50 mg of blank GP-MS suspended in 2 ml of 0.9% saline. The treatment schedule is displayed in Table 2. GP-MS with a 40% crosslinking degree were selected since a peritoneal residence time of 14 days is ensured^32^.

**Table 2:** Different intraperitoneal treatment groups and their paclitaxel dose in mg per kg body weight.

Treatment	PTX dose (mg/kg)	Number of mice
0.9% saline	0	11
Blank GP-MS	0	11
PTX_EtOH_ GP-MS	7.5	10
PTX_nano_ GP-MS	7.5	11
PTX_nano_ GP-MS	35	11
Nab-PTX	35	12

Animals were continuously evaluated from the day of treatment until occurrence of death, attainment of predefined endpoints or until the end of the study (90 days post-treatment). General conditions (activity, feces evaluation, behavior pattern and other clinical signs), body weight and mortality were carefully monitored. Mice were euthanized when they met following humane endpoints: excessive weight loss (body weight loss of ≥20% at any timepoint or ≥15% maintained for 72 h compared with pre-treatment weight), presence of ascites and abnormal behavior.

Weekly, bioluminescence imaging (BLI) was performed for tumor growth follow-up of each mouse. Mice were injected IP with 0.2 ml of D-luciferin (15 mg/ml; PerkinElmer, Zaventem, Belgium) 10 minutes prior to BLI. After 10 min, bioluminescence images were acquired by the IVIS Lumina II system (PerkinElmer). Luminescence was quantified using the Living Image^®^ 4.3.1 software (PerkinElmer).

**Figure 1:**
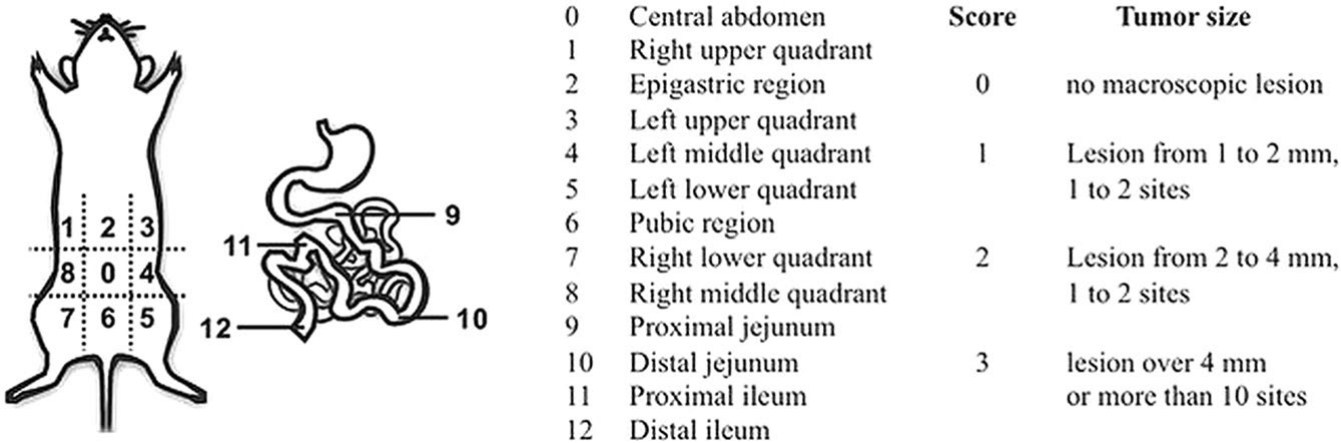
Peritoneal carcinomatosis index (PCI) in mice. Visual exploration of the 13 standard regions of the abdominal cavity for PCI scoring was carried out using magnifiers (copy from Derrien A. *et al*.^87^).

In case of death, after euthanasia when humane endpoints were reached or at the final day of the study, a midline laparotomy was performed, and the abdominal cavity was exposed and photographed. Each mouse was assigned a Peritoneal Carcinomatosis Index (PCI) score. The abdominal cavity was divided in 13 standard regions as described by Sugarbaker^38^ and adapted for rodents by Klaver^39^. For each region a score from 0 to 3 was given based on size and quantity of tumor nodules (Fig. 1). Furthermore, an ascites score was given of 0 (no ascites present) or 1 (ascites present) to all animals. Survival time of each mouse was assessed. The therapeutic efficacy was measured as an increase in overall survival time compared to no treatment (i.e. 0.9% saline). Increase in life span (ILS) was calculated as median survival time treatment minus median survival time control, % ILS was calculated as (median survival time treatment/median survival time control *100) minus 100.

Samples were taken from cecum, jejunum, peritoneum/abdominal wall, liver, pancreas, spleen, and if applicable, tumor for histopathological examination. All samples were fixed immediately by immersion in 4% paraformaldehyde in PBS for 72 h and embedded in paraffin. The tissues were then serially sectioned and stained with hematoxylin and eosin (H&E). Slides were analyzed in a blinded manner and microscopic images were taken with a light microscope (ColorView I, BX43F, Olympus, Tokyo, Japan).

Immunohistochemical staining was performed with Paired Boxed Gene 8 (PAX-8) polyclonal antibody (Proteintech), dilution 1/400, using an automatic immunostainer (Benchmark XT; Ventana Medical Systems, Oro Valley, AZ, USA), according to the manufacturer’s instructions. Visualization for all antibodies was achieved with the ultraView Universal DAB Detection kit (Ventana Medical Systems).

Statistical analysis was performed using Graphpad Prism^TM^ 5.2 (Graphpad Software, Inc.; La Jolla, CA, USA). *In vivo* data were analysed using non-parametric tests (Mann-Whitney-U, Kruskall-Wallis and Dunn’s method). Survival analysis was estimated using the Kaplan-Meier method and survival differences between groups were evaluated using the log-rank test. A p-value < 0.05 was considered to indicate statistical significance.

### Pharmacokinetics of PTX-treatments

Blood microsamples were taken daily from the tail vein until 14 days post-treatment. 10 µl blood was spotted on a blood spot card (PerkinElmer 226 Bioanalysis RUO Card, Perkin Elmer, Greenville, USA). PTX concentration of dried blood spot (DBS) samples was determined by a previously developed UPLC-MS/MS methode^40^. The lower limit of quantification of PTX in a DBS sample was 1 ng/ml. The method imprecision was less than 14.6% and bias was less than ±11.9%.

Numerical deconvolution was performed using Phoenix WinNonlin^®^ IVIVC Toolkit 7.0 (Certara LP, St. Louis, Missouri, USA) to evaluate the percent of drug absorbed *in vivo* from the PTX-formulations.

The individual predicted blood and peritoneal PTX profiles were obtained from a population pharmacokinetic (PK) model, carried out in NONMEM^®^ (version 7.3, Icon Development Solutions, Ellicott City, MD, USA). PK parameters, peak concentration (C_max_), time-to-peak concentration (t_max_) and area under the curve (AUC), were predicted from the PK model.

A pharmacokinetic-pharmacodynamic (PKPD) model was developed in NONMEM^®^ using the predicted peritoneal concentrations as the time-varying input for the effect of PTX-treatments on survival outcome and blood concentrations were considered to be responsible for toxicity-induced death. Details of the PKPD model are described in Fie X. *et al*.^41^.

### Evaluation of ccCK18 as a biomarker in ovarian cancer

During the survival study, blood of the different PTX treatment and control groups was analyzed to determine the concentration of caspase-cleaved cytokeratin 18 (ccCK18) in order to evaluate its potential as a PD biomarker to monitor treatment response in microscopic peritoneal disease of ovarian origin. Blood samples were taken twice a week from the tail vein during the survival study and animals were pooled in time. Blood was centrifuged for plasma collection. Apoptosis-associated ccK18 concentrations were quantitatively determined in plasma using a solid-phase sandwich enzyme immunoassay, M30 Apoptosense^®^ enzyme-linked immunosorbent assay (PEVIVA^®^; VLVbio, Sundbyberg, Sweden) in accordance with the manufacturer’s instructions. The detection limit of the assay was 25 U/l.

### Ethics approval

All animal experiments were approved by the Animal Ethics Committee of the Faculty of Medicine at Ghent University (ECD 17/83).

## Results

### Incorporation of paclitaxel into genipin-crosslinked gelatin microspheres

In earlier work, GP-MS were developed for IP administration, and their applicability to prevent post-operative peritoneal adhesions was demonstrated^32^. Based on the slow degradation rate of GP-MS (as a function of the crosslinking degree) these systems could also be applied to provide sustained intraperitoneal delivery of a chemotherapeutic agent. Therefore, the GP-MS were loaded with PTX. After crosslinking the GP-MS, they were loaded with PTX via immersion in a PTX-containing medium. Since a hydrophilic environment is preferred for this drug loading step to allow GP-MS swelling and to maximize PTX penetration into GP-MS, PTX was dissolved in a 75/25 *v/v* ethanol/water mixture to allow partial swelling of the microspheres and to provide sufficient solubility of PTX in the loading medium. The highest absolute amount of PTX was incorporated into GP-MS using a loading solution with the highest PTX concentration. However, incorporation efficiency reduced as a function of the PTX concentration in the loading solution (Table 3). At higher crosslinking degree less PTX was incorporated in the microspheres (Table 4) as drug diffusion into GP-MS is probably physically hindered due to the intramolecular network of genipin^32^. Overall, the PTX load of the GP-MS remained low. Incorporation of PTX was improved when GP-MS were loaded by immersion in an aqueous PTX nanosuspension (Table 3). The PTX nanocrystals had a particle size of 283.7 ± 1.2 nm and a polydispersity index of 0.16. The PTX load in these GP-MS was threefold higher compared to the procedure with an ethanolic PTX solution. Since the PTX nanocrystals were diluted in physiological saline, GP-MS could maximally swell during incorporation and hence diffusion of PTX into the microspheres is enhanced.

**Table 3:** Influence of PTX concentration (1, 2.5 and 5 mg/ml) and type of loading medium (ethanolic solution vs. nanosuspension) on PTX load and incorporation efficiency in GP-MS (n = 6).

PTX conc. in loading medium (mg/ml)	PTX load (µg) per mg MS	Incorporation efficiency (%)
**PTX-ethanolic solution**		
1	1.1 ± 0.2	10.8 ± 2.1
2.5	1.4 ± 0.2	5.5 ± 0.7
5	3.4 ± 0.6	6.8 ± 1.2
**PTX-nanosuspension**		
1	4.4 ± 0.7	43.6 ± 7.3
2.5	6.2 ± 0.5	24.7 ± 1.9
5	9.9 ± 0.8	19.7 ± 1.6

Microspheres with a crosslinking degree of 40% were used.

**Table 4:** Influence of crosslinking degree on PTX load and incorporation efficiency in GP-MS (n = 6).

Crosslinking degree (%)	PTX load (µg) per mg MS	Incorporation efficiency (%)
7	4.2 ± 1.8	8.5 ± 3.6
25	4.0 ± 1.6	8.0 ± 3.1
40	3.4 ± 0.6	6.8 ± 1.2
60	2.5 ± 0.4	4.9 ± 0.8
70	1.8 ± 0.2	3.6 ± 0.5

Microspheres were loaded with a 5 mg/ml PTX-ethanolic solution.

By adjusting the settings during the loading procedure (microsphere mass, volume and type of loading medium, concentration PTX in nanosuspension), the amount of PTX encapsulated in the microspheres could be easily varied, with a maximum PTX load of 39.2 ± 6.5 µg PTX/mg MS (Table 5). Varying the MS crosslinking degree yielded similar PTX loads for the PTX nanosuspension (data not shown), while the effect of this parameter was more pronounced when GP-MS were loaded with a PTX-ethanolic solution, indicating that the type of loading medium determines the efficiency of PTX incorporation in GP-MS.

**Table 5:** PTX load (µg) per mg GP-MS (n = 6) and incorporation efficiency in MS (n = 6).

Mass GP-MS (mg)	Volume loading medium (ml)	PTX conc. in nanosuspension (mg/ml)	PTX load (µg) per mg MS	Incorporation efficiency (%)
100	2	1	12.3 ± 3.3	61.5 ± 16.7
		2.5	18.9 ± 1.3	37.8 ± 2.6
		5	28.7 ± 13.1	28.7 ± 13.1
50	2	1	16.6 ± 1.6	41.4 ± 4.1
		2.5	30.5 ± 7.8	30.5 ± 7.8
		5	39.2 ± 6.5	19.6 ± 3.3
200	3	1	10.7 ± 1.8	71.3 ± 12.1
		2.5	16.5 ± 1.8	44.0 ± 4.8
		5	27.6 ± 3.9	36.8 ± 5.3

GP-MS mass, loading medium volume and PTX concentration in nanosuspension were varied during the incorporation procedure. Microspheres with a crosslinking degree of 40% were used.

### *In vitro* PTX release from paclitaxel-loaded GP-MS

**Figure 2:**
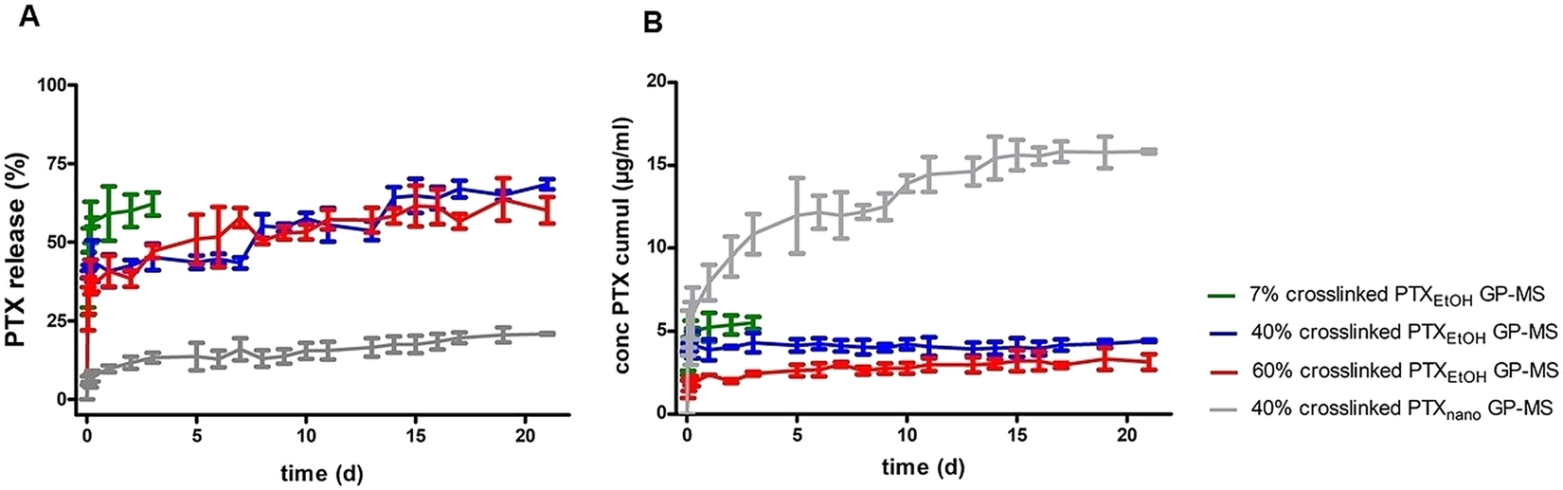
Total cumulative PTX percentage (**A**) and PTX concentration (**B**) released as a function of time from GP-MS with different degrees of crosslinking (7, 40 and 60% for green, blue and red line, respectively) loaded with a 5 mg/ml PTX-ethanolic solution or from GP-MS with 40% crosslinking loaded with a 5 mg/ml PTX-nanosuspension (grey line).

Cumulative release of PTX from PTX-GP-MS is calculated as the sum of PTX and 7-epi PTX. PTX has a complex structure with several hydrolytically sensitive ester groups and a chiral center that readily undergoes epimerization. Due to its instability in an aqueous environment, the stereoisomer 7-epi-PTX was abdundantly detected in drug release samples. No other PTX degradation products were present. Stereoisomer 7-epi PTX is thermodynamically stable and its mechanism of action is identical to PTX^42–45^. Drug release profiles are displayed in Fig. 2.

GP-MS loaded with an ethanolic PTX solution and PTX nanosuspension showed both a sustained PTX release over three weeks. Drug release from PTX_EtOH_-GP-MS shows a biphasic profile with an initial burst release during the first day followed by a sustained release. After 3 days 62.0 ± 4.0% PTX is released from 7% crosslinked PTX_EtOH_-GP-MS, and visual inspection showed that these microspheres were degraded. No significant differences in release profile were observed between PTX_EtOH_ microspheres with a 40 and 60% crosslinking degree.

A drug release of 19.0 ± 0.8% was reached from PTX_nano_-GP-MS (40% crosslinked) after 21 days. A 7% burst release from PTX_nano_-GP-MS was seen during day 1 and PTX release was sustained during the entire drug release study. It has been reported that PTX nanocrystals exhibit a slower and more sustained release than Taxol^® ^^46^ and the incorporation of PTX nanocrystals into microspheres will further control the release rate of PTX.

The absolute amount of PTX released from GP-MS was highest from microspheres loaded using the PTX nanosuspension (Fig. 2B) since more PTX could be incorporated into those microspheres. However, percentage drug release was higher for microspheres loaded with PTX ethanolic solution (Fig. 2A). This can partly be linked to PTX solubility in the release medium. Tween 80 was added to the release medium to increase PTX solubility, and literature reports solubility values of PTX in PBS with 0.1% Tween 80 in a 6 to 11 µg/ml range^47,48^. While the maximum PTX concentrations in release medium of PTX_EtOH_-GP-MS are within this range (8.4, 6.8 and 4.9 µg/ml for 7, 40 and 60% crosslinked microspheres, respectively), complete PTX release from PTX_nano_-GP-MS would largely exceed reported solubility values (78.4 µg PTX/ml in release medium). However, *in vitro* dissolution will be indicative for *in vivo* behaviour since sink condition will also not be reached *in vivo* because of the limited peritoneal fluid volume.

### Influence of PTX-loaded GP-MS on ovarian cancer cell viability

Susceptibility of OC cells to PTX-GP-MS was determined based on ATP quantification by CellTiter Glo 2.0 Luminescent assay. In general, all PTX-GP-MS decreased viability of the cell lines in a time- and concentration-dependent manner, in order of sensitivity: SK-OV-3 Luc IP1 < SK-OV-3 < OVCAR-3. Since SK-OV-3 Luc IP1 cells are *in vivo* selected to mimic more aggressive disseminated cells^34^, higher IC_50_ values are expected. After 24 hours treatment, each cell type retained at least half of its viability. IC_50_ values after a treatment duration of 72 and 168 h were calculated to compare sensitivity of OC cells to the test formulations (Table 6). IC_50_ values of each test formulation were in the nanomolar range. Comparable IC_50_ values for OC cells exposed to PTX were found in literature although it should be noted that each value is influenced by the specific experimental conditions^49–52^. Antitumoral activity of PTX-GP-MS increased by prolonging exposure time since PTX is gradually released from the GP-MS. In comparison, antitumoral activity of a PTX solution is faster, reflected in significantly lower IC_50_ values when compared to PTX-GP-MS in all cell lines after 72 h incubation. Over time, both PTX-GP-MS and PTX solution will eradicate OC cells with non-significant differences in IC_50_ values after 168 and 336 h incubation. Limited to no cell viability is observed after 336 h exposure to PTX treatment. Additionally, comparison of GP-MS with the same degree of crosslinking (40%) with a different type of loading medium showed that GP-MS loaded with a PTX nanosuspension displayed significantly lower IC_50_ values for the SK-OV-3 (Luc IP1) cell line. PTX nanosuspension is stabilized by Pluronic^®^ F127 polymer. It is known that Pluronic block copolymers have cytotoxicity-promoting properties. They can sensitize tumor cells to cytostatic drugs and are able to inhibit multiple mechanisms of drug resistance in multi drug resistance (MDR) cancer. The chemosensitizing effect is most pronounced in MDR cancer cell lines, but it is also observed in regular cancer cells including SK-OV-3 cells^13,53,54^. This was not observed for OVCAR-3 cells since their susceptibility to PTX is higher and IC_50_ values were similar for all PTX treatments.

**Table 6:** IC_50_ values of PTX after SK-OV-3 (Luc IP1) and OVCAR-3 cells were treated during 72 and 168 h with PTX-GP-MS and PTX-solution.

Cell line	7% crossl PTX_EtOH_-GP-MS	25% crossl PTX_EtOH_-GP-MS	40% crossl PTX_EtOH_-GP-MS	60% crossl PTX_EtOH_-GP-MS	40% crossl PTX_nano_-GP-MS	PTX-solution
	IC_50_ values (nM) after 72 h treatment					
SK-OV-3	28.1 ± 1.9	14.9 ± 2.2	18.3 ± 2.3	16.9 ± 1.8	8.6 ± 1.0	5.9 ± 0.9
OVCAR-3	8.2 ± 1.8	11.4 ± 2.2	12.1 ± 1.8	6.3 ± 1.8	9.5 ± 1.3	4.1 ± 1.8
SK-OV- 3 Luc IP1	38.0 ± 2.3	27.1 ± 2.0	23.2 ± 2.0	16.6 ± 2.3	3.5 ± 2.4	7.1 ± 2.1
**Cell line**	**IC50 values (nM) after 168 h treatment**					
SK-OV-3	15.0 ± 2.7	7.9 ± 1.8	22.9 ± 2.2	7.2 ± 1.9	4.9 ± 1.8	7.5 ± 1.9
OVCAR-3	4.7 ± 1.7	6.3 ± 1.7	9.5 ± 1.8	4.5 ± 1.7	7.1 ± 1.7	4.1 ± 1.7
SK-OV-3 Luc IP1	11.2 ± 6.0	37.2 ± 2.3	16.1 ± 2.0	30.3 ± 2.3	10.3 ± 1.5	25.9 ± 1.9

Mean IC_50_ and SD of triplicates are shown.

### Efficacy of PTX-microspheres in a microscopic peritoneal carcinomatosis xenograft model

**Figure 3:**
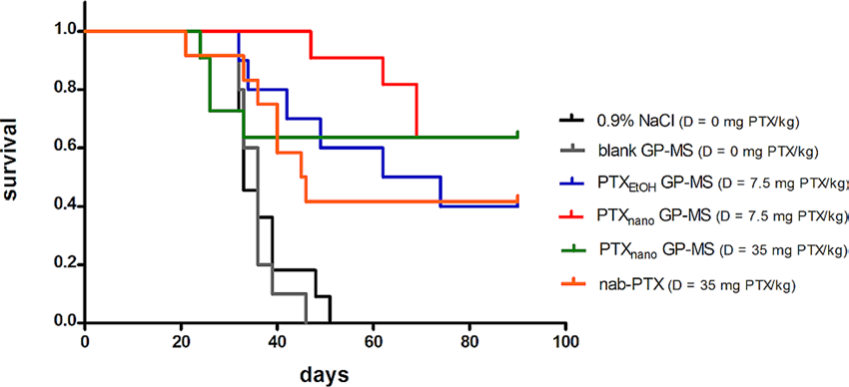
Kaplan-Meier survival curve of mice treated with control (0.9% saline, blank GP-MS) or PTX-formulation (PTX_EtOH_-GP-MS (D = 7.5 mg PTX/kg), PTX_nano_-GP-MS (D = 7.5 or 35 mg PTX/kg) or nanoparticular albumin-bound PTX, nab-PTX (D = 35 mg/kg)). Mice treated with a PTX-formulation lived significantly longer than mice treated with a control in a microscopic peritoneal disease xenograft model.

Survival curves of control and PTX-treated animals are displayed in Fig. 3. Subsequent survival comparison using the log-rank test for trend showed a significant inferior survival of control animals (0.9% saline and blank GP-MS-treated mice, p < 0.05) compared to animals who received a treatment with a PTX formulation. Median survival time is displayed in Table 7.

**Table 7:** Median survival time (MST) of mice after IP administration of different treatments in a microscopic peritoneal carcinomatosis xenograft model and observed increase in life span (ILS) compared to control animals receiving an IP injection of 0.9% saline.

Treatment	Dose PTX (mg/kg)	MST (d)	ILS (d)	%ILS
0.9% saline	0	33	NA	NA
blank GP-MS	0	36	NA	NA
PTX_EtOH_ GP-MS	7.5	68	35	106.1
PTX_nano_ GP-MS	7.5	90	57	172.7
PTX_nano_ GP-MS	35	90	57	172.7
nab-PTX	35	45.5	12.5	37.9

NA = non-applicable; nab-PTX = nanoparticular albumin-bound paclitaxel.

A median survival of 33 and 36 days was observed after IP administration of 0.9% saline and blank GP-MS, respectively. There was no significant difference in survival outcome between both control groups. IP administration of a PTX treatment after IP xenograft inoculation significantly increased survival of the animals. However, there were no significant differences in survival outcome observed between the different treatment groups. A median survival of 68 days was seen after IP treatment with PTX_EtOH_-GP-MS dosed at 7.5 mg PTX/kg with ILS of 35 days compared to untreated animals. At the final day of the study, 40.0% of the PTX_EtOH_-GP-MS-treated animals remained alive. Following IP administration of PTX_nano_-GP-MS, dosed at 7.5 and 35 mg PTX/kg, 63.6% of the PTX_nano_-GP-MS-treated mice of both dosage groups were still alive upon completion of the survival study. The median survival time was not reached for PTX_nano_-GP-MS. An ILS of at least 57 days compared to untreated animals was noticed. It should be remarked that the events of death happened at earlier time points for the high-dosed PTX_nano_-GP-MS group (day 24, 26 and 33 post-treatment), whereas in the low-dosed group, deaths occurred at day 47, 62 and 69 post-treatment. It is possible that the deaths in the high-dosed PTX_nano_-GP-MS were treatment-related, while they were related to progression of the disease in the low-dosed group. In contrast to the pronounced survival prolongation due to the slow release of PTX from PTX-GP-MS and thus the longer residence time of PTX in the abdominal cavity, an ILS of 12.5 days and %ILS of 37.9% was achieved after bolus injection of nab-PTX (35 mg PTX/kg). 41.7% of the nab-PTX treated animals remained alive until completion of the study.

**Figure 4:**
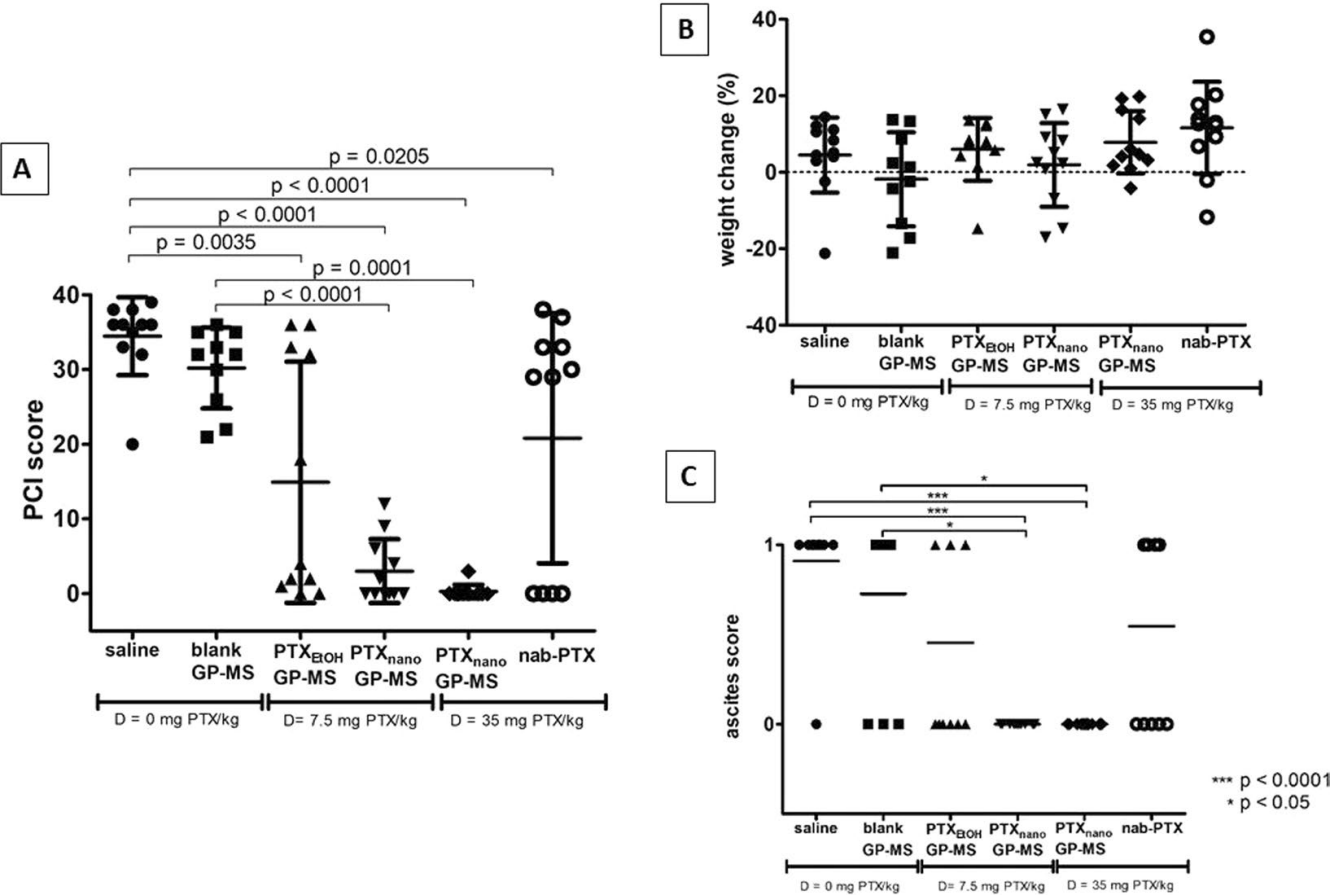
Antitumor efficacy of control groups (0.9% saline (n = 11) and blank GP-MS (n = 11)) and treatment groups (PTX_EtOH_-GP-MS (D = 7.5 mg PTX/kg, n = 10), PTX_nano_-GP-MS (D = 7.5 or 35 mg PTX/kg, n = 11/group) and nanoparticular albumin-bound PTX, nab-PTX (D = 35 mg PTX/kg, n = 12)) in microscopic peritoneal disease xenografts, based on (**A**) peritoneal carcinomatosis index (PCI), (**B**) percentage weight change and (**C**) ascites score. Bars indicate median and interquartile range.

PCI scores of the control and drug-treated mice are plotted in Fig. 4A. Comparison of the PCI scores of the different treatment groups demonstrated that there were no significant differences between the two control groups or between the PTX-treatment groups. Significant differences in PCI score were observed when comparing PCI scores of mice treated with 0.9% saline versus mice treated with PTX_EtOH_-GP-MS (p = 0.0035); versus PTX_nano_-GP-MS (p < 0.0001, both dose groups) and versus nab-PTX (p = 0.0205). Also, significant differences in PCI score were seen when comparing blank GP-MS and PTX_nano_-GP-MS dosed at 7.5 mg PTX/kg (p = 0.0001) and dosed at 35 mg PTX/kg (p < 0.0001). When comparing PCI scores of blank GP-MS-treated mice to the scores of mice treated with PTX_EtOH_-GP-MS or nab-PTX, no significant differences could be observed.

**Figure 5:**
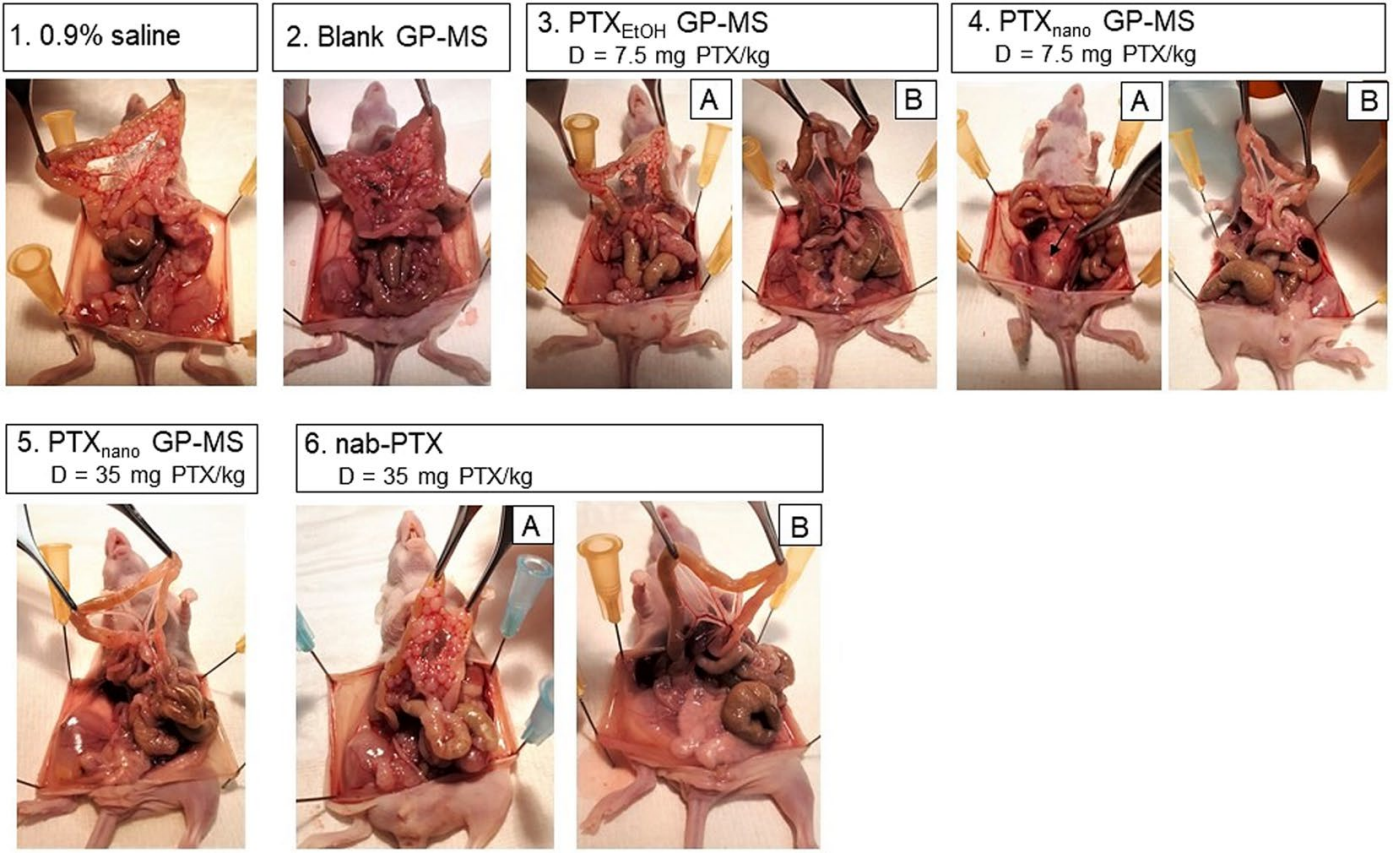
Macroscopic images of intraperitoneal dissemination. Control animals (0.9% saline (1) and blank GP-MS (2) display peritoneal carcinomatosis. PTX_EtOH_-GP-MS treated animals (3) either developed peritoneal carcinomatosis (3A) or remained disease-free (3B). PTX_nano_ GP-MS treated animals either developed in a few cases a local tumor node (4A, arrow) or remained disease free (4B, 5). Animals treated with nanoparticular albumin-bound PTX, nab-PTX, either developed peritoneal carcinomatosis (6A) or not (6B).

High PCI scores of 34.4 ± 2.2 and 30.2 ± 5.4 were assigned to the 0.9% saline and blank GP-MS treated animals, respectively. Numerous small tumor nodules were found in the abdominal cavity at the visceral peritoneum and mesentery, and tumor infiltration into liver, spleen, stomach and diaphragm was also observed (Fig. 5-1 and 5-2). Omental metastases were found in 90.9% and 80.0% of the mice in the 0.9% saline and blank GP-MS group, respectively.

Whereas all control mice developed peritoneal carcinomatosis (PC) in a similar manner, cancer did not progress in the same way in the PTX-treated mice. Treatment with PTX_EtOH_-GP-MS was successful to eradicate microscopic disease and to prevent tumor progression in 4 of the 10 animals (Fig. 5-3A). However, despite a lack of PC, tumor growth was seen in 3 of the 4 animals at the site of injection. During removal of the injection needle from the peritoneal space, a remainder of the SK-OV-3 Luc IP1 cell suspension was possibly released in the subperitoneal space between the skin and abdominal muscle layer. Because of the potency of the cell line after *in vivo* selection this could result in a local tumor node despite a low number of cells inoculated^34^. Five of the PTX_EtOH_-GP-MS-treated animals developed PC (Fig. 5-3B). Three animals died from disease progression post-treatment day 32, 34 and 49, similar to the survival of control animals. The treatment had no effect on microscopic peritoneal disease. Two animals died at day 62 and 74 post-treatment. In these animals, sustained PTX release from PTX_EtOH_-GP-MS prolonged survival, but it was insufficient to prevent recurrence of peritoneal disease. In one animal, a large tumor developed dorsally while the typical pattern of numerous small peritoneal nodules was not observed.

Seven of the PTX_nano_-GP-MS-treated animals, dosed at 7.5 mg PTX/kg, did not develop PC and no other local tumors were detected (Fig. 5-4B). Sustained release of PTX from GP-MS loaded with a PTX nanosuspension was effective to eliminate microscopic disease and could prevent recurrence of peritoneal disease during the period of the survival study. While one animal grew a local tumor subperitoneally following a spill of tumor cells during withdrawal of the needle after IP injection for tumor inoculation, it did not develop peritoneal carcinomatosis. Three of the eleven animals died because of large dorsal tumor nodules, as illustrated in Fig. 5-4A. The typical IP dissemination pattern of peritoneal disease was not observed in those animals, but the large dorsal tumor mass was correlated with cachexia with a weight loss of 7, 15 and 17%. The other animals of this treatment group gained weight. PTX_nano-_GP-MS treated animals, dosed at 35 mg PTX/kg, displayed no PC upon autopsy, including the four animals who died before completion of the study. Only 1 animal developed a local tumor node subperitoneally.

**Figure 6:**
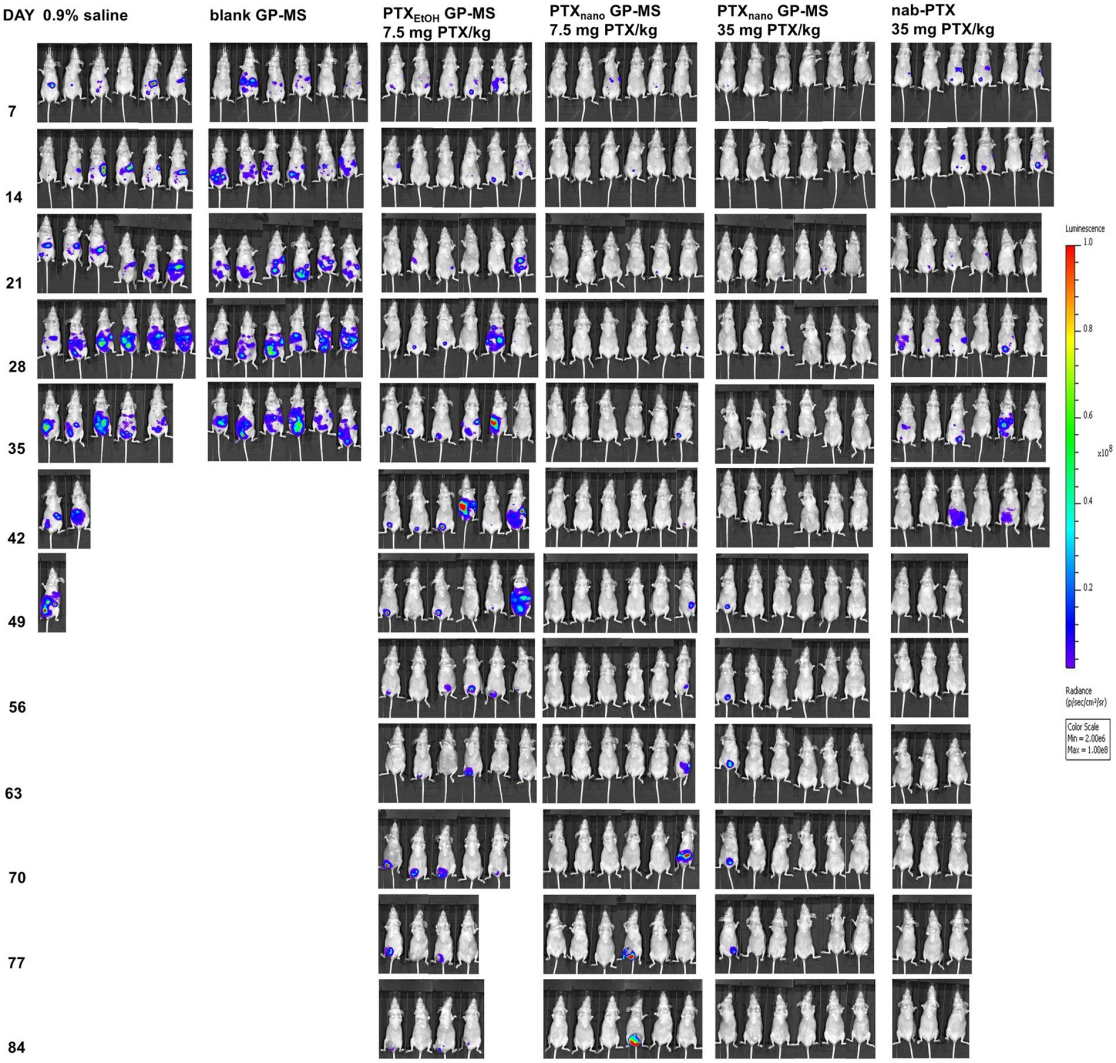
Representative *in vivo* bioluminescence images of IP tumor burden in different control (IP injection of 0,9% saline or blank GP-MS) and treatment groups (IP injection of PTX_EtOH_-GP-MS (D = 7.5 mg PTX/kg), PTX_nano_-GP-MS (D = 7.5 mg PTX/kg or D = 35 mg PTX/kg), nanoparticular albumin-bound PTX, nab-PTX (D = 35 mg PTX/kg)).

The nab-PTX-treated mice could be divided into responders and non-responders. The mice either developed peritoneal carcinomatosis with a high PCI score at the time of autopsy (58.3% of the animals, Fig. 5,6A) or a single injection of a high dose of the immediate release nab-PTX formulation was efficient for the prophylaxis of PC recurrence (41.7% of the animals, Fig. 5,6B).

When comparing the weight change of the mice (Fig. 4B), no significant differences could be observed between the groups. No unambiguously conclusions can be derived from weight change over time since an increase in weight is not always related to an efficacious treatment. While extreme weight loss is evidently related to disease progression, ascites - which increases the body weight - is associated with peritoneal metastases and a poor prognosis^55^.

Ascites scores of each mouse per group are plotted in Fig. 4C. Ascites was present in 90.9% and 70.0% of the mice who received an IP injection of 0.9% saline or blank GP-MS, respectively. Treatment with PTX_nano_-GP-MS, at both dosages, significantly decreased incidence of ascites compared with animals treated with 0.9% saline (p = 0.0016) and blank GP-MS (p = 0.0082). None of the PTX_nano_-GP-MS treated mice had ascites present at the time of death or at the end of the study. No significant differences in ascites score were observed when control groups were compared with PTX_EtOH_-GP-MS or nab-PTX treated animals. Ascitic fluid was observed in 50.0 and 54.5% of PTX_EtOH_-GP-MS and nab-PTX treated mice, respectively. Additionally, the incidence of ascites was significantly lower for animals treated with PTX_nano_-GP-MS compared with animals treated with PTX_EtOH_-GP-MS (p = 0.0190) or nab-PTX (p = 0.0143).

BLI images of representative mice are displayed in Fig. 6. A longitudinal follow-up of tumor burden and effect of the applied therapies on microscopic PC was possible during the survival study. BLI signals of the individual mice of each group during the survival study are displayed in Suppl. Fig. 1.

An increase of BLI signal over time was seen in all control animals, related to the progression of peritoneal carcinomatosis. Maximal total flux of 1.9 × 10^9^ and 1.85 × 10^9^ p/s was measured in animals treated with 0.9% saline and blank GP-MS, respectively. Follow-up of the BLI signals of drug-treated animals allowed to individually monitor the efficacy of the administered treatment. An effective response of PTX treatment over time was seen when BLI signal remained low (baseline signal, total flux of 10^4^–10^5^ p/s) and constant during the entire study due to eradication of initial microscopic peritoneal disease. When the BLI signal increased, the treatment failed to (further) prevent relapse of peritoneal disease. As seen in the PTX_EtOH_-GP-MS and nab-PTX groups, when the proton flux started to increase, the animal started to develop PC with maximal total flux of 3.42 × 10^9^ and 7.44 × 10^8^ p/s, respectively. Similarly, the increase of total flux in some animals of the PTX_nano_-GP-MS group, indicated the presence of a tumor with a maximal measured total flux of 3.52 × 10^9^ p/s. Combination of the BLI images and semi-quantitative proton flux data allowed for a careful monitoring of disease progression and evaluation of the extent of disease. Data of the total flux alone do not provide information about the localization of the tumor(s). When analyzing BLI images, it is clear that the animals in the PTX_EtOH_-GP-MS-group where proton flux increased, had a widespread distribution of tumor cells in the abdominal cavity, while animals with increasing BLI signal in the PTX_nano_-GP-MS group, dosed at 7.5 mg/kg, showed a localized tumor growth. Additionally, shifts of BLI signal can be used as an indication of the optimal time point to re-administer the therapy.

### Histopathology and toxicity

**Figure 7:**
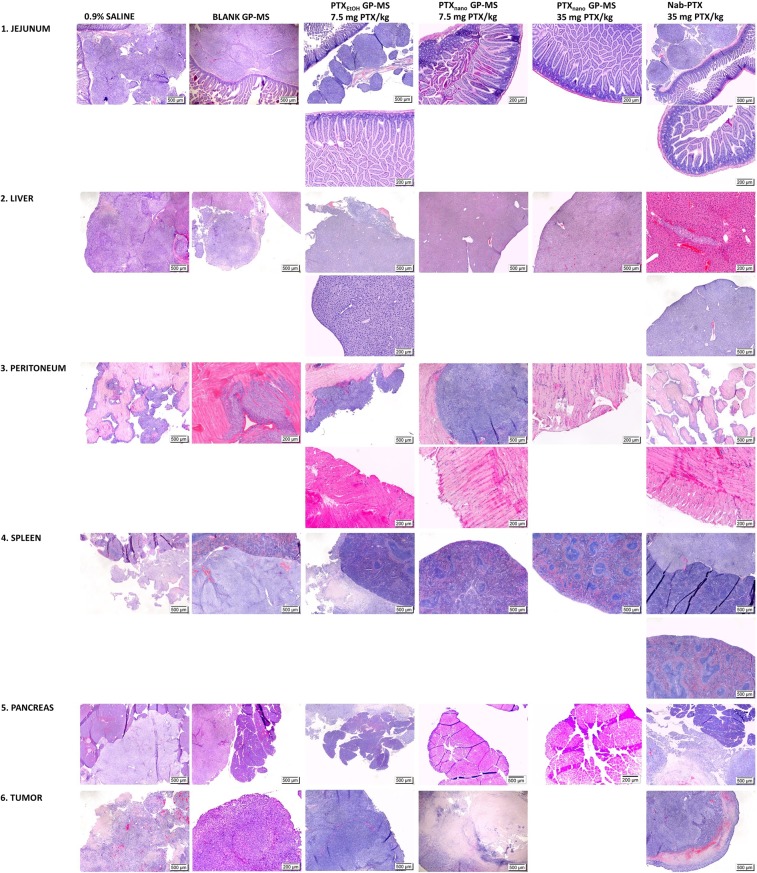
Optical micrographs of H&E-stained sections of jejunum, liver, peritoneum, spleen, pancreas and tumor of untreated (0.9% saline, blank GP-MS) or PTX-treated (PTX_EtOH_- or PTX_nano_-GP-MS and nanoparticular albumin-bound PTX, nab-PTX) mice in a microscopic peritoneal carcinomatosis xenograft model of ovarian origin.

Histopathological analysis of the major abdominal organs and peritoneal tissue was performed to evaluate the extent of peritoneal disease and possible local toxicity of the different treatments. Microphotographs of H&E-stained tissues of the mice of different treatment groups are displayed in Fig. 7.

**Figure 8:**
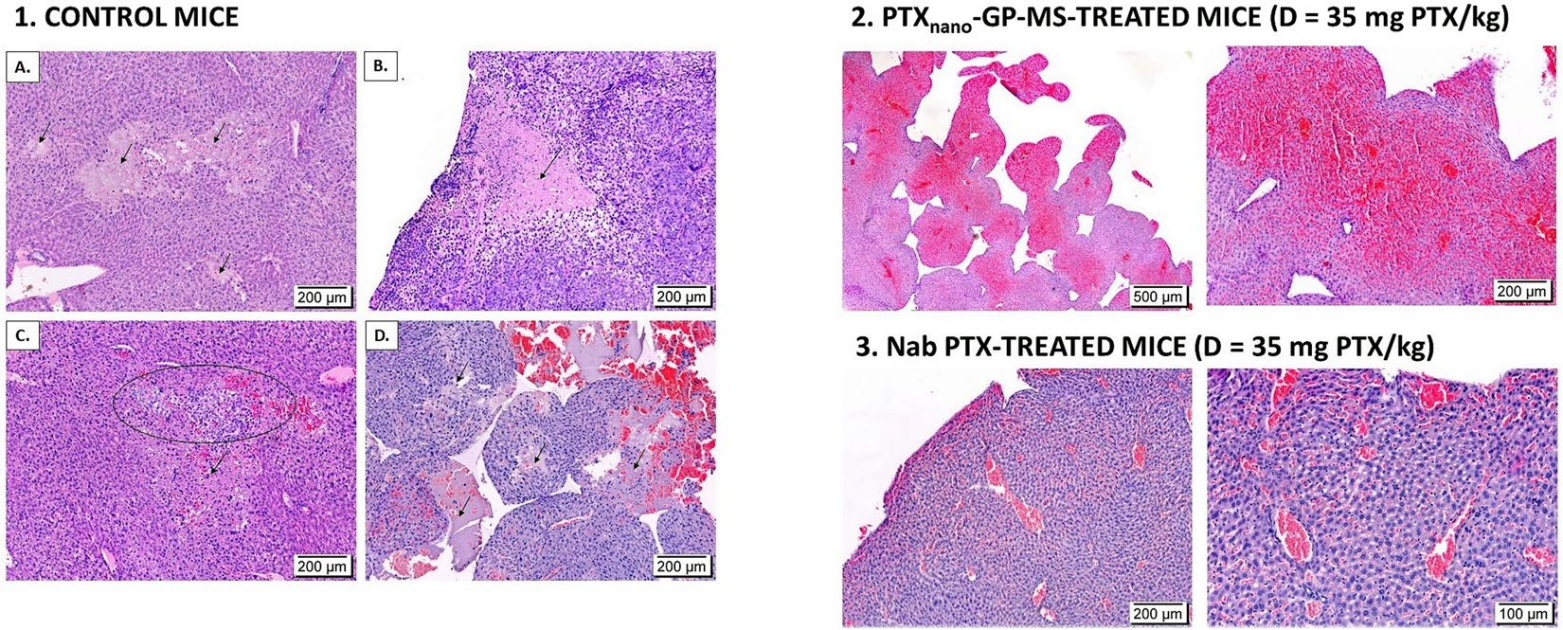
Optical micrographs of liver irregularities of H&E-stained slices of livers of control, PTX_nano_-GP-MS or nab-PTX-treated mice (D = 35 mg PTX/kg). Liver tissue of control mice (1A–D) displayed areas of necrosis, indicated by an arrow. Tumor cells infiltrated liver parenchyma (1C). Massive infiltration of SK-OV-3 Luc IP1 cells eventually leads to hemorrhage and peliosis hepatis (1D). A case of sinusoidal obstruction syndrome was observed in a PTX_nano_-GP-MS treated mice (2) and sinusoidal congestion was also noticed in nab-PTX-treated mice.

Numerous tumor nodules consisting of actively dividing cells were clearly visible in the mesentery of the jejunum in control mice. It is known that malignant cells quickly and efficiently invade the mesentery because of its unique microvasculature^56^. Morphology of the jejunum of control mice was normal with no signs of inflammation or tumor cells in the mucosa, indicating that the tumor cells only invaded visceral peritoneal tissue. Liver tissue of control mice not only showed tumor cells at the capsule of the liver, but tumor cells had also infiltrated the liver parenchyma (Fig. 8-1C). The gall bladder of control animals was also metastasized with tumor cells as illustrated in the photomicrograph of a liver of a mouse treated with 0.9% saline in Fig. 7. When peritoneal disease had extended into the liver, several liver disorders were seen as displayed in Fig. 8. Necrotic hepatic disease was observed (1A-D). This phenomenon is typically seen in rapidly growing hepatic metastasis, causing hypoxia and subsequently necrosis. Rupture of tumor blood vessels in necrotic areas may result in massive hemorrhage and peliosis hepatis^57^ (Fig. 8-1D). Parietal peritoneum of control mice showed active inflammation of mesothelium with the presence of tumor cells of ovarian origin as confirmed by a positive PAX-8 staining (Suppl. Fig. 2). In most cases, peritonitis and tumor development remained localized at the mesothelium, but in a few cases extension of the inflammation into muscle tissue of the abdominal wall was observed (blank GP-MS). Large tumor nodules were present in the adipose tissue of the omentum and splenoportal fat of control mice. They are known preferential sites for tumor cell attachment because of their vascularization and the presence of immune aggregates, ‘milky spots’^56^. Tumor tissue of control mice was vascularized and consisted of actively dividing tumor cells, but also regions of necrosis were observed, typically seen in locally advanced solid tumors. Prolonged hypoxia, imbalance between oxygen consumption and supply, can cause necrosis. Reasons for hypoxia in tumors include an abnormal structure and function of microvessels supplying the tumor, a longer diffusion distance between blood vessels and tumor cells and a lower oxygen transport capacity of blood^58,59^.

Positive PAX-8 staining of the tissues of control mice confirmed that tumor cells were of ovarian origin (Suppl. Fig. 2).

H&E staining (Fig. 7) of jejunum tissue of mice treated with PTX_EtOH_-GP-MS showed either the presence of metastases of ovarian origin (positive PAX-8 staining, Suppl. Fig. 2) in the mesentery or no metastases in case of response to the treatment. In both cases, the architecture of the small intestines showed no irregularities or abnormalities. When the treatment was ineffective, liver metastases were found at the surface of the liver in combination with infiltration of tumor cells into liver parenchyma with hepatic necrotic disease. Liver tissue of mice who responded to the PTX_EtOH_-GP-MS-treatment displayed a normal morphology. In the mesothelial lining of the peritoneum covering the abdominal wall, more inflammatory and tumor cells of ovarian origin were detected, as confirmed by a positive PAX-8 staining. However, when the release of PTX from PTX_EtOH_-GP-MS was effective to treat microscopic peritoneal disease, the peritoneum and skeletal muscle of the abdominal wall displayed a normal morphology. Similar to the control mice, omental metastases were found in mice who did not respond to PTX_EtOH_-GP-MS treatment. A dense, solid tumor was observed and tumor cells were attached to the richly vascularized adipose tissue of omental tissue associated to spleen and pancreas, without affecting the architecture of the organs itself.

The high response rate to the PTX_nano_-GP-MS treatment, dosed at 7.5 mg/kg PTX, was reflected upon histological examination of abdominal organ tissues. No tumor cells or abnormalities were seen in tissues of jejunum, liver, spleen and pancreas. As observed via BLI imaging and during autopsy, some mice developed a local tumor nodule at the site of injection, which was confirmed upon histopathological examination. A large dense tumor node invaded the mesothelial lining of the peritoneum into the skeletal muscle of the abdominal wall. Blood vessels were present in the tumor nodule. PAX-8 expression confirmed that the tumor cells were of ovarian origin. Additionally, evaluation of tumor tissue of the 3 mice who developed a large dorsal tumor showed extended regions of tumor necrosis. This can be either attributed to the typical observations of large localized tumor nodules where the imbalance between oxygen supply and use results in necrosis.

Histopathological observation of H&E stained tissues of mice who received PTX_nano_-GP-MS treatment, dosed at 35 mg PTX/kg, confirmed that there was no peritoneal carcinomatosis present. Tissues of small intestines, peritoneum, pancreas and spleen had a normal appearance. However, while most of the liver tissue showed no abnormalities, liver tissues of 3 animals clearly confirmed sinusoidal dilatation with congestive central veins (i.e. sinusoidal obstruction syndrome) and the presence of peliosis hepatis. The syndrome was especially pronounced in one animal (Fig. 8-2). Little is known about the effects on the liver of a prolonged IP PTX release. Sinusoidal congestion was observed in liver tissue of high dose PTX_nano_-GP-MS treated mice who died prior to the end of the study, whereas liver tissue of mice who remained alive until the end of the study did not show abnormalities. It is possible that the mice died of treatment-related toxicity because of the prolonged exposure to a higher concentration of PTX, resulting in a high portal vein PTX concentration and higher hepatoxicity, since no hepatoxicity was noticed when mice were treated with PTX_nano_-GP-MS dosed at 7.5 mg PTX/kg.

IP treatment with a bolus injection of 35 mg/kg nab-PTX resulted in either an effective treatment of microscopic peritoneal disease, which was reflected by a normal histological appearance of jejunum, liver, peritoneum and spleen, or the immediate release of nab-PTX appeared to be ineffective. This latter was reflected by tumor nodules in the mesentery of jejunum and ileum, infiltration of liver parenchyma and capsule with tumor cells (as confirmed by positive PAX-8 staining), omental metastases with tumor cells - in the connective and adipose tissue associated to the spleen and tumor cells in the mesothelium of the parietal peritoneum. The tumor tissue consisted of a dense population of tumor cells, with blood vessels present and also regions of tumor necrosis. Two cases showed a congestive liver with sinusoidal obstruction, which could be either attributed to tumor infiltration into the liver or to the high chemotherapy dose.

### Pharmacokinetics of PTX-treatments

**Figure 9:**
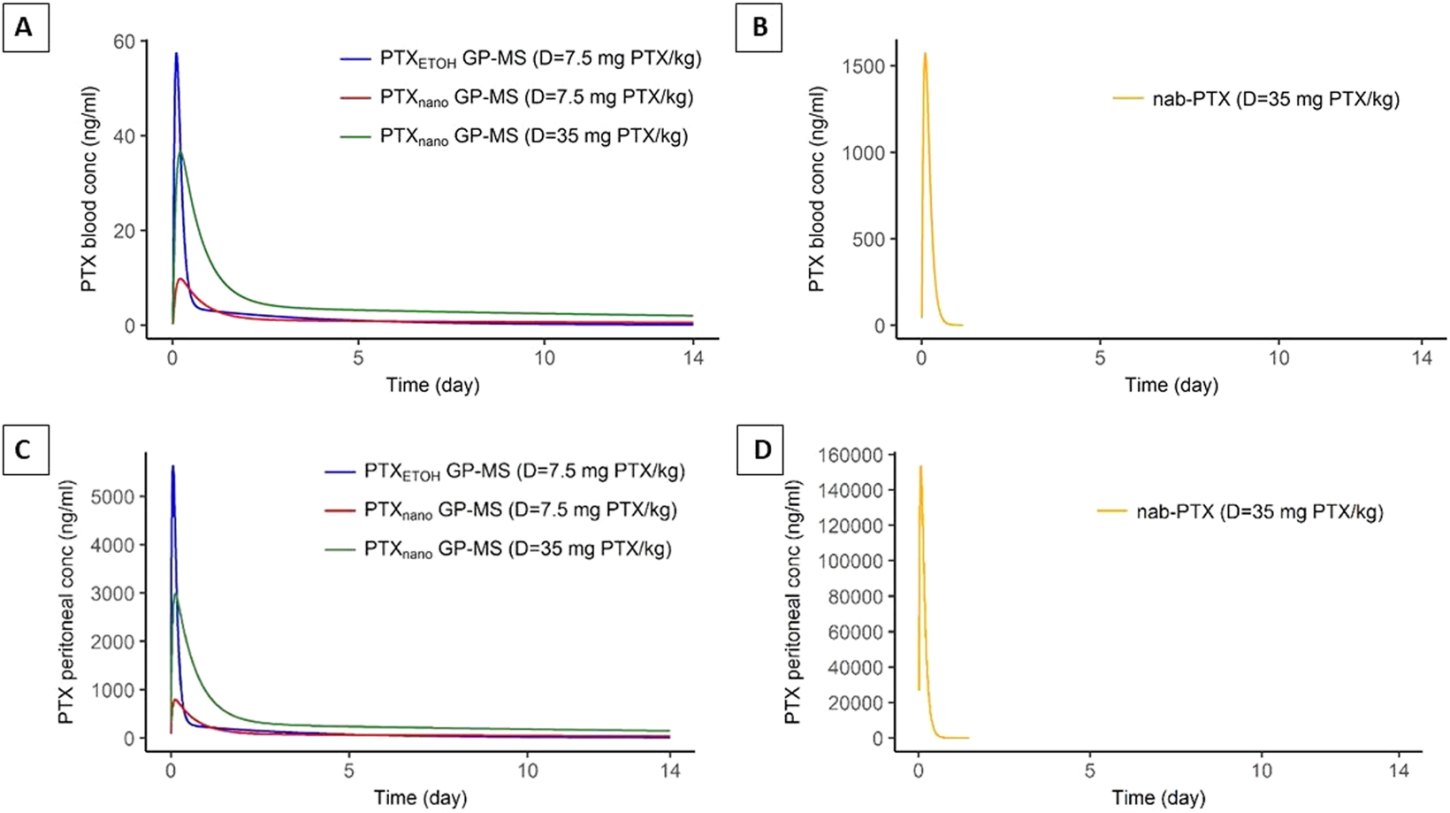
Average predicted PTX concentration (ng/ml) as a function of time after IP administration of different types of PTX-GP-MS (PTX_EtOH-_GP-MS at a dose of 7.5 mg PTX/kg and PTX_nano_-GP-MS at doses of 7.5 and 35 mg PTX/kg) in blood (**A**) and peritoneal fluid (**C**) of mice. PTX concentrations were detectable up to 14 days. In contrast, (**B,D**) represent the predicted average PTX concentration after a single IP injection of nanoparticular albumin-bound PTX, nab-PTX (D = 35 mg PTX/kg) in blood and peritoneal fluid, respectively.

Average systemic and average predicted local peritoneal PTX concentrations after IP administration of the different PTX treatments (PTX-GP-MS and nab-PTX) as a function of time are displayed in Fig. 9. A burst release was observed during the first day after IP injection of PTX-GP-MS followed by a sustained release of PTX from PTX-GP-MS over the period of the *in vivo* drug release study, resulting in a prolonged systemic exposure to PTX but at a low concentration level. Higher local PTX concentrations were obtained compared to systemic PTX concentrations because of the prolonged peritoneal PTX release from the GP-MS and a slow systemic absorption.

Pharmacokinetic parameters of average systemic and peritoneal PTX concentrations are displayed in Table 8. Burst release was higher after IP administration of PTX_EtOH_-GP-MS compared to PTX_nano_-GP-MS: C_max_ in blood and peritoneal fluid were respectively 5.9 and 7.1-fold higher for PTX_EtOH_-GP-MS compared to PTX_nano_-GP-MS at a dose of 7.5 mg PTX/kg. Remarkably, peak peritoneal and blood concentration of PTX released from PTX_EtOH_-GP-MS (D = 7.5 mg PTX/kg) were also higher when compared to PTX_nano_-GP-MS, at a dose of 35 mg PTX/kg. A dose-proportional increase of AUC_0-∞_ and C_max_ was seen when the dose was escalated to 35 mg PTX/kg, indicating linear pharmacokinetics.

**Table 8:** Pharmacokinetic parameters of average systemic and peritoneal PTX concentration-time profiles: area under the curve (AUC), peak concentration (C_max_) and time to peak concentration (t_max_) of the different IP treatment groups

Treatment	PTX dose (mg/kg)	AUC_0–14d_ (µg.h/ml)	AUC_0-∞_ (µg.h/ml)	C_max_ (µg/ml)	t_max_ (h)
**Average systemic PTX concentrations**					
PTX_EtOH_-GP-MS	7.5	0.6	0.7	0.06	2.5
PTX_nano_-GP-MS	7.5	0.4	0.7	0.01	4.9
PTX_nano_-GP-MS	35	1.6	3.1	0.04	5.0
Nab-PTX	35	9.1	10.7	1.6	2.5
**Average peritoneal PTX concentrations**					
PTX_EtOH_-GP-MS	7.5	46.8	49.8	5.6	1.5
PTX_nano_-GP-MS	7.5	31.7	52.9	0.8	3.05
PTX_nano_-GP-MS	35	119.5	203.1	3.0	3.05
Nab-PTX	35	676.9	677.1	153.6	1.5

Nab-PTX = nanoparticular albumin-bound PTX.

Nab-PTX, the nanoparticulate formulation of PTX, was unable to sustain the PTX release over a prolonged period of time which is reflected in its different PK profile. PTX was rapidly absorbed from the peritoneal cavity into the systemic circulation, while GP-MS released PTX more gradually. 24 hours after IP injection of nab-PTX, 54% of the PTX-dose was systemically absorbed. In contrast, only 7 and 4% PTX was systemically absorbed 24 hours after IP administration of PTX_EtOH_-GP-MS and PTX_nano_-GP-MS, respectively. After 14 days, approximately 10–15% of the PTX amount released from GP-MS was systemically absorbed. Additionally, since the PTX dose is rapidly released after IP administration of nab-PTX, significantly higher peak concentrations were observed compared to the slow PTX release from PTX_nano_-GP-MS at the same dose, both systemically and locally. The higher percentage absorption and high systemic PTX peak concentration may result in systemic side effects such as neutropenia.

Individual peritoneal PTX profiles are displayed in Suppl. Fig. 3. Despite between-animal variability in peak PTX concentration, a higher burst release was not the predictive factor for differences in treatment response. In the PTX_EtOH_-GP-MS and nab-PTX-treated mice, there were either ‘treatment-responders’ when microscopic peritoneal disease was successfully eradicated, and PC did not recur, or ‘treatment-non-responders’ when PC did develop. However, no significant differences were found in peak peritoneal PTX concentrations of ‘treatment-responders’ and ‘treatment-non-responders’ within the PTX_EtOH_-GP-MS and nab-PTX-groups.

### Evaluation of ccCK18 as a biomarker in ovarian cancer

**Figure 10:**
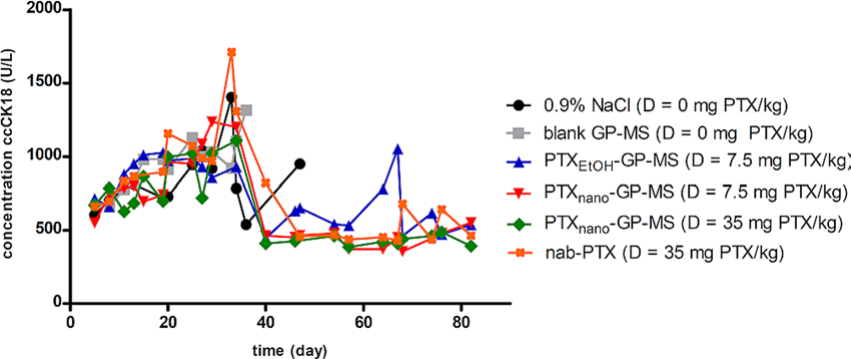
Average concentration of ccCK18 (U/L) as a function of time measured in plasma samples of different control (IP injection of 0,9% saline or blank GP-MS) and treatment groups (IP injection of PTX_EtOH_-GP-MS (D = 7.5 mg PTX/kg), PTX_nano_-GP-MS (D = 7.5 mg PTX/kg or D = 35 mg PTX/kg), nanoparticular albumin-bound PTX, nab-PTX (D = 35 mg PTX/kg)) in a human xenograft microscopic peritoneal carcinomatosis mouse model.

Average concentrations of caspase-cleaved cytokeratin 18 (ccCK18) over time measured in plasma of mice treated with the different therapies (control, PTX-GP-MS and nab-PTX) are shown in Fig. 10.

An increase of ccCK18 concentration was observed for the control animals related to disease progression. Thus, the development of peritoneal carcinomatosis could be monitored via the evolution of ccCK18 in time.

Monitoring of ccCK18 levels of the PTX treatment groups showed elevated ccCK18 levels during the first 30 days. In case of the PTX_EtOH_-GP-MS and nab-PTX treated mice, this can be explained in two ways. Treatment non-responders developed peritoneal carcinomatosis and elevated ccCK18 levels were seen because of spontaneous apoptosis of tumor cells. On the other hand, elevated ccCK18 concentrations were also observed in treatment-responders because of PTX-induced apoptosis of SK-OV-3 IP1 Luc cells. After absorption of PTX from the abdominal cavity, ccCK18 returns to a baseline level. Individual ccCK18 profiles of representative mice treated with PTX_EtOH_-GP-MS are displayed in Suppl. Fig. 4 where it is clearly seen that animals 7 and 11 were non-responders, and animals 1, 4 and 8 treatment-responders. Animal 5 showed an increase of ccCK18 after 58 days, which can be linked to a relapse of peritoneal disease after initial treatment response.

All PTX_nano_-GP-MS-treated animals had elevated ccCK18 levels during the first month due to drug-induced apoptosis of SK-OV-3 IP1 Luc cells caused by the local prolonged PTX release from the PTX_nano_-GP-MS. PTX was measurable up to 14 days in blood, but *in vitro* studies demonstrated that PTX-microspheres were able to sustain PTX concentrations up to 21 days. After 1 month, PTX will be completely absorbed from the peritoneal cavity and ccCK18 levels return to a baseline level until completion of the survival study.

Previous studies have shown that there is a significant correlation between ccCK18 and drug-induced toxicity, e.g. damage to normal tissue. However, because the M30 Apoptosense^®^ assay is human specific and should only detect ccCK18 released by human xenograft tumor cells, interference due to cell death of normal epithelial tissues of mice should be limited^60^.

## Discussion

Randomized controlled clinical trials have shown that the addition of IP chemotherapy to the first-line treatment cytoreductive surgery and platinum-taxane based IV chemotherapy increased overall survival and disease-free progression of patients with advanced ovarian cancer^1,2,17^. Nevertheless, prognosis of the disease remains poor due to a high percentage of recurrence. Approximately 80% of advanced OC patients will relapse within 5 years. It is assumed that the high rate of recurrence can be attributed to the short residence time of chemotherapeutics in the abdominal cavity^1,3,8,18,20^. Therefore, a post-surgical adjuvant IP treatment able to sustain an anticancer agent in the abdomen for weeks or even months is of high clinical interest. Therefore, we evaluated PTX-GP-MS as a novel IP drug delivery system.

Our *in vitro* data suggest that PTX_nano_-GP-MS are most promising for IP drug delivery. The highest amount of PTX could be incorporated into GP-MS when GP-MS were immersed in a PTX-nanosuspension. Also, PTX loading could be most easily varied, offering a flexible therapeutic strategy. Subsequently, released PTX concentrations were highest both during *in vitro* and *in vivo* release. Additionally, the sustained release profile of PTX_nano_-GP-MS was more favorable compared to PTX_EtOH_-GP-MS.

*In vivo* results showed that PTX-treatment significantly improved survival of mice in a microscopic peritoneal carcinomatosis mouse model. No significant differences in survival outcome were observed between the different PTX-treatment groups. The survival study was ended 90 days post-treatment, an increase of the study period could be required to obtain more conclusive results. However, mice treated with IP PTX_nano_-GP-MS, at both dose levels, increased the life span with at least 57 days compared to an increase of 35 and 12.5 days following IP treatment with PTX_EtOH_-GP-MS (D = 7.5 mg PTX/kg) and nab-PTX (D = 35 mg PTX/kg), respectively. Additionally, IP treatment with PTX_nano_-GP-MS significantly decreased PCI score compared to both control groups, whilst no differences of PCI score could be observed when comparing PTX_EtOH_-GP-MS and nab-PTX-treated animals to animals treated with blank GP-MS. Furthermore, treatment with PTX_nano_-GP-MS significantly decreased the presence of ascites compared to mice treated with blank GP-MS and 0.9% saline. In contrast, the presence of ascites did not differ after an IP treatment with PTX_EtOH_-GP-MS or nab-PTX compared to the controls. These results support that PTX_nano_-GP-MS seem to present a promising and effective post-surgical adjuvant IP treatment strategy.

A prolonged residence of PTX in the abdominal cavity clearly improved treatment outcome and progression free survival of mice compared to a single IP injection with nab-PTX. Despite a high C_max_ of 154 µg/ml in peritoneal fluid following IP administration of nab-PTX, the formulation is quickly absorbed from the peritoneal cavity with a high systemic peak concentration. After one day, PTX was not detectable anymore in blood or peritoneal fluid. In contrast, PTX remained detectable both in blood and peritoneal fluid up to 14 days following IP treatment with PTX-GP-MS. Although both PTX_EtOH_- and PTX_nano_-GP-MS were able to prolong the residence time of PTX in the abdominal cavity, PTX_nano_-GP-MS were more efficacious in eradicating minimal peritoneal disease and preventing recurrent peritoneal carcinomatosis. It has already been reported that PTX nanocrystals demonstrate a high cellular uptake due to their size and accumulate in tumor tissue, increasing the efficacy of PTX therapy^13,61,62^.

As systemic absorption of a chemotherapeutic agent from a local drug depot system is limited, systemic toxicity will be largely avoided. But little is known about the influence of a prolonged residence time of a chemotherapeutic agent on normal tissues and organs of the abdominal cavity. Three mice (27%) receiving PTX_nano_-GP-MS at a 35 mg PTX/kg dose showed liver toxicity (sinusoidal dilation and congestive veins in liver parenchyma), while no abnormalities were seen in other abdominal organs and peritoneal tissue. Additionally, hematological analysis showed no deviations of white blood cell count, absolute neutrophil count, platelets and other parameters. This hepatoxicity can be a complication of chemotherapy as it is known that several chemotherapeutic agents have a toxic effect on the liver, inducing injuries on hepatic venous endothelium and consequently the sinusoidal obstruction syndrome. This phenomenon is most frequently described following administration of 5-fluorouracil, cyclophosphamide and oxaliplatin and is rarely seen in paclitaxel therapy^63–66^. However, elevated liver function values (bilirubin, alkaline phosphatase (ALP), aspartate and alanine aminotransferase (AST and ALT, respectively)) were reported in patients receiving IV PTX therapy, although the liver values rarely largely exceeded the upper limits of normal ALP, AST or ALT values and are dose dependent^67–73^. Liver injury is a direct effect of PTX since the molecule is predominantly metabolized by the liver^74^. Morbidity and adverse effects reported during IP treatment with PTX are mostly abdominal pain and hematological and neurologic toxicity^1,17,75^. Although an increase in hepatic injury was reported by Armstrong *et al*., the peritoneal clearance of paclitaxel might have been altered because the drug was given after IP cisplatin^2^. The prolonged peritoneal exposure to a higher PTX concentration might have resulted in a high portal vein PTX concentration, and higher hepatotoxicity since no hepatotoxicity was observed when mice were treated with PTX_nano_-GP-MS dosed at 7.5 mg PTX/kg. Monitoring of liver values and portal vein concentrations will be recommended and prophylactic administration of anti-oxidants or glutathione could overcome the toxicity syndrome^76^.

A pharmacokinetic-pharmacodynamic (PKPD) model accounting for the positive drug treatment effect and unwanted toxicity of PTX-treatment on survival of mice in a microscopic peritoneal carcinomatosis model was developed (unpublished model based analysis). Dose simulations of PTX_nano_-GP-MS from the developed PKPD model demonstrated that the optimal dose of PTX_nano_-GP-MS to achieve a good survival outcome with minimal drug-related toxicity ranged from 7.5 to 15 mg PTX/kg. Sustained PTX release in a low dose is thus effective to prevent recurrence of peritoneal disease, thereby avoiding local toxic side-effects because of the prolonged presence of PTX in the abdominal cavity.

Finally, this work evaluated ccCK18 as a biomarker to monitor drug-response and efficacy. Cytokeratin-18, a protein present in epithelial cells, is cleaved by caspases during apoptosis. The resulting fragments, caspase-cleaved cytokeratin-18, are release into the blood^77–79^. Longitudinal follow-up of ccCK18 concentration in serum or plasma could be interesting to monitor treatment response since the anticancer activity of PTX can be directly related to the induction of apoptosis of tumor cells^77,79^. Furthermore, the correlation between drug-induced tumor cell apoptosis and an increase in ccCK18 has already been proven^80–82^.

Elevated ccCK18 levels were observed during the first month of the survival study, indicating drug-induced apoptosis of tumor cells. An increase of ccCK18 is expected when PTX induces mitotic arrest and apoptosis of SK-OV-3 IP1 Luc cells, thereby demonstrating the potential of ccCK18 as a predictive biomarker for treatment efficacy. However, interpretation of ccCK18 is complex because release of ccCK18 can be a response to various stimuli including treatment response, drug-induced toxicity and increase of tumor burden. A correlation was observed between elevated levels of ccCK18 and tumor burden. It has already been shown that patients with epithelial carcinomas have elevated levels of (cc)CK18 related to the spontaneous apoptosis of tumor cells. Higher levels of ccCK18 have been associated to the number of involved organs, performance status and shorter median survival^60^. Nevertheless, a positive correlation between increased ccCK18 levels and treatment response was observed in this study and in several other (pre)clinical trials^60,77,78,81^. Therefore, the inclusion of ccCK18 in a biomarker panel (cancer antigen 125, human epididymis protein 4, e-cadherin,…)^83–85^ in ovarian cancer therapy should not be neglected.

Our preclinical results in mice are promising for further investigation of the effectiveness of PTX-GP-MS in the treatment of peritoneal carcinomatosis of ovarian origin. Low-dosed PTX_nano_-GP-MS seem to be most promising postsurgical IP adjuvant formulation with a significant improvement of survival, little systemic absorption and no toxicity is observed while maintaining a sustained release of PTX over 14 days. Prior to a first-in-human phase I trial, pharmacology and toxicology studies should be performed in a rodent and non-rodent species and the most sensitive species will be chosen for safe starting dose determination taking into account a conversion factor^86^.

## Conclusion

This study in a human xenograft microscopic peritoneal carcinomatosis mouse model showed that prolonging the residence time of PTX in the abdominal cavity is an effective way to improve treatment outcome of advanced ovarian cancer. PTX_nano_-GP-MS was the most effective formulation to eradicate microscopic peritoneal carcinomatosis and to prevent recurrent peritoneal disease. Life span of mice improved with a significant reduction of tumor burden based on PCI score, BLI imaging and ascites incidence compared to control animals. In contrast, no significant differences in PCI score and presence of ascites were observed when comparing PTX_EtOH_-GP-MS and nab-PTX treated animals to control animals. PTX_nano_-GP-MS seem to be a promising postsurgical adjuvant IP depot system. Previous work already demonstrated the effectiveness of GP-MS to prevent peritoneal adhesions in a mouse model, a common complication after CRS. The promising preclinical results and the potential dual application of the concept in the treatment modality of advanced ovarian cancer mark their clinical relevance and encourage further (pre)clinical evaluation of the formulation.

## Supplementary information

Supplementary Figures

## Data Availability

The datasets used and/or analysed during the current study are available from the corresponding author on reasonable request.
