# Supplementary material for: Preclinical evaluation of local prolonged release of paclitaxel from gelatin microspheres for the prevention of recurrence of peritoneal carcinomatosis in advanced ovarian cancer
Source: Sci Rep. 2019 Oct 16;9:14881. doi: 10.1038/s41598-019-51419-y (PMC6795903; doi:10.1038/s41598-019-51419-y)
Supplement: Supplementary file 1 — Supplementary Figures [file 41598_2019_51419_MOESM1_ESM.docx]

**Preclinical evaluation of local prolonged release of paclitaxel from gelatin microspheres for the prevention of recurrence of peritoneal carcinomatosis in advanced ovarian cancer**

Kaat De Clercq^1,7^, Feifan Xie^2^, Olivier De Wever^3,7^, Benedicte Descamps^4^, Anne Hoorens^5^, An Vermeulen^6^, Wim Ceelen^6,7^, Chris Vervaet^1*^

^1^Laboratory of Pharmaceutical Technology

Ghent University, Ottergemsesteenweg 460, 9000 Ghent, Belgium

[Kaat.DeClercq@UGent.be](mailto:Kaat.DeClercq@UGent.be)

^2^Laboratory for Medical Biochemistry and Clinical Analysis

Ghent University, Ottergemsesteenweg 460, 9000 Ghent, Belgium

[Feifan.Xie@UGent.be](mailto:Feifan.Xie@UGent.be)

[AnMC.Vermeulen@UGent.be](mailto:AnMC.Vermeulen@UGent.be)

^3^Laboratory of Experimental Cancer Research

Ghent University, Corneel Heymanslaan 10, 9000 Ghent, Belgium

[Olivier.DeWever@UGent.be](mailto:Olivier.DeWever@UGent.be)

^4^Infinity (IBiTech-MEDISIP)

Department of Electronics and Information Systems

Ghent University, Corneel Heymanslaan 10, 9000 Ghent, Belgium

[Benedicte.Descamps@UGent.be](mailto:Benedicte.Descamps@UGent.be)

^5^Department of Pathology

Ghent University Hospital, Corneel Heymanslaan 10, 9000 Ghent, Belgium

[Anne.Hoorens@uzgent.be](mailto:Anne.Hoorens@uzgent.be)

^4^Department of Gastro-intestinal Surgery

Ghent University Hospital, Corneel Heymanslaan 10, 9000 Ghent, Belgium

[Wim.Ceelen@UGent.be](mailto:Wim.Ceelen@UGent.be)

^7^Cancer Research Institute Ghent (CRIG)

Corresponding author:

Professor Chris Vervaet

Laboratory of Pharmaceutical Technology

Faculty of Pharmaceutical Sciences

Ottergemsesteenweg 460, 9000 Gent, Belgium

[Chris.Vervaet@UGent.be](mailto:Chris.Vervaet@UGent.be)

T: +32 9 264 80 69


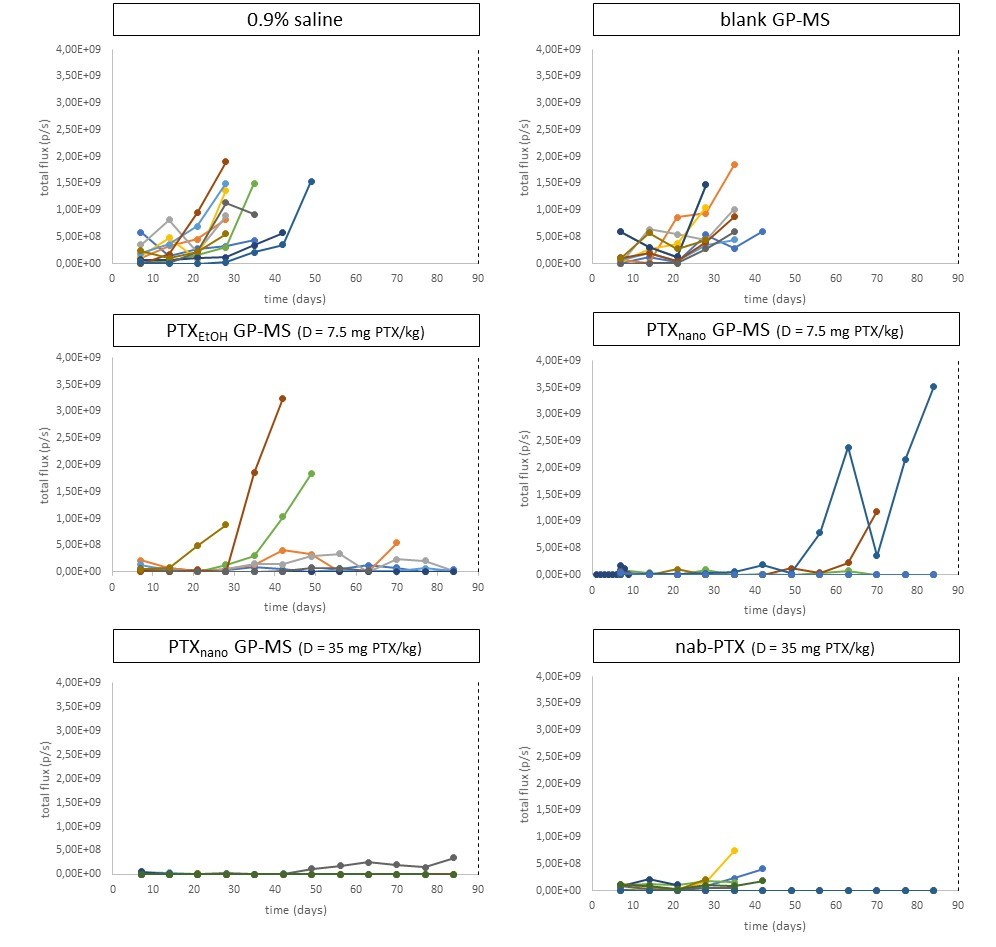


**Suppl. Figure 1.** Bioluminescence imaging signals (total proton flux) in mice with luciferase positive peritoneal carcinomatosis xenografts after IP treatment with controls (0.9% saline or blank GP-MS) or paclitaxel treatment (PTX_EtOH_-GP-MS, PTX_nano_-GP-MS, nanoparticular albumin-bound PTX (nab-PTX))


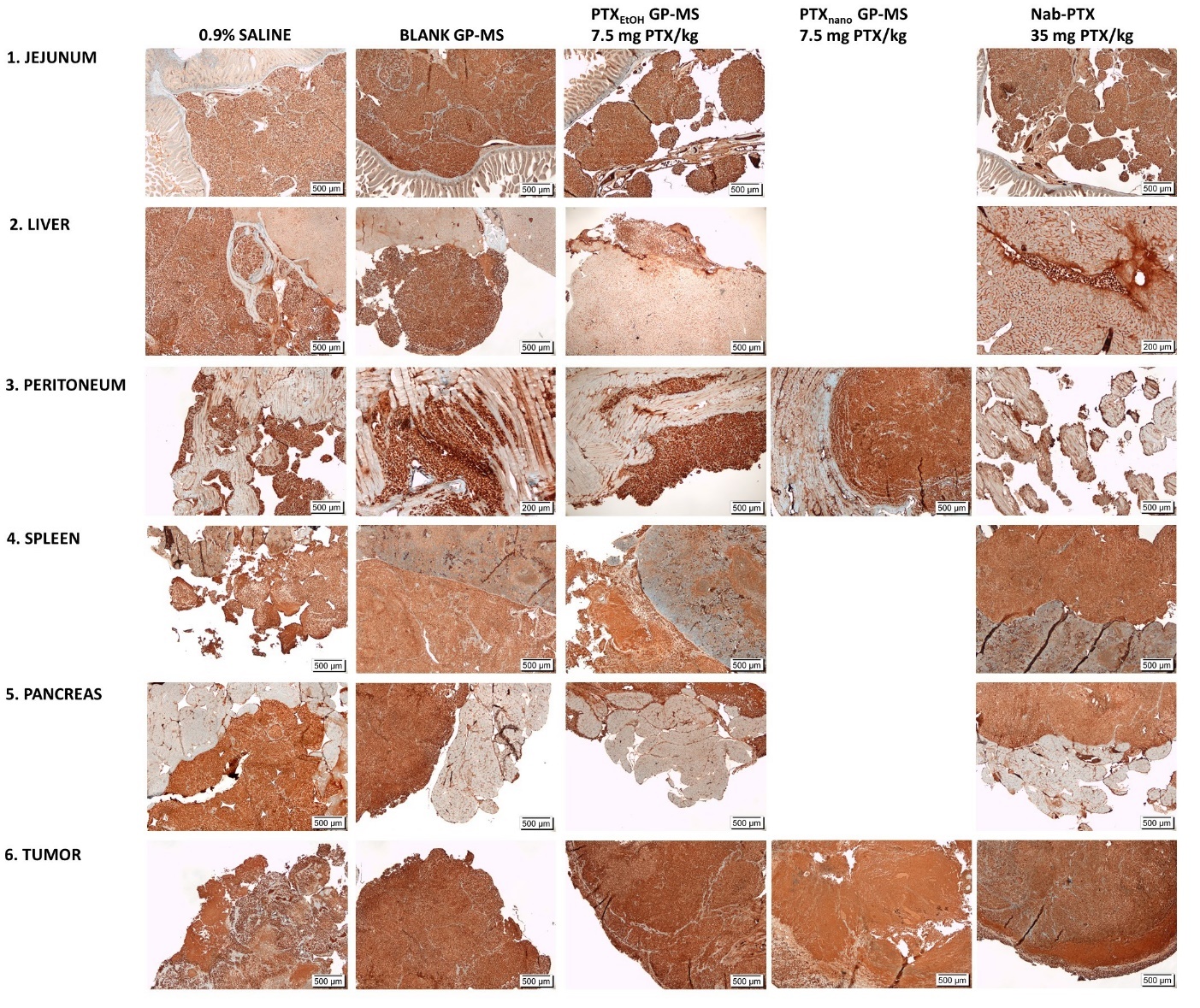


**Suppl Figure 2.** Optical micrographs of PAX-8-stained sections of jejunum, liver, peritoneum, spleen, pancreas and tumor of untreated (0.9% saline, blank GP-MS) or PTX-treated (PTX_EtOH_- or PTX_nano_-GP-MS and nab-PTX) mice in a microscopic peritoneal carcinomatosis xenograft model of ovarian origin.


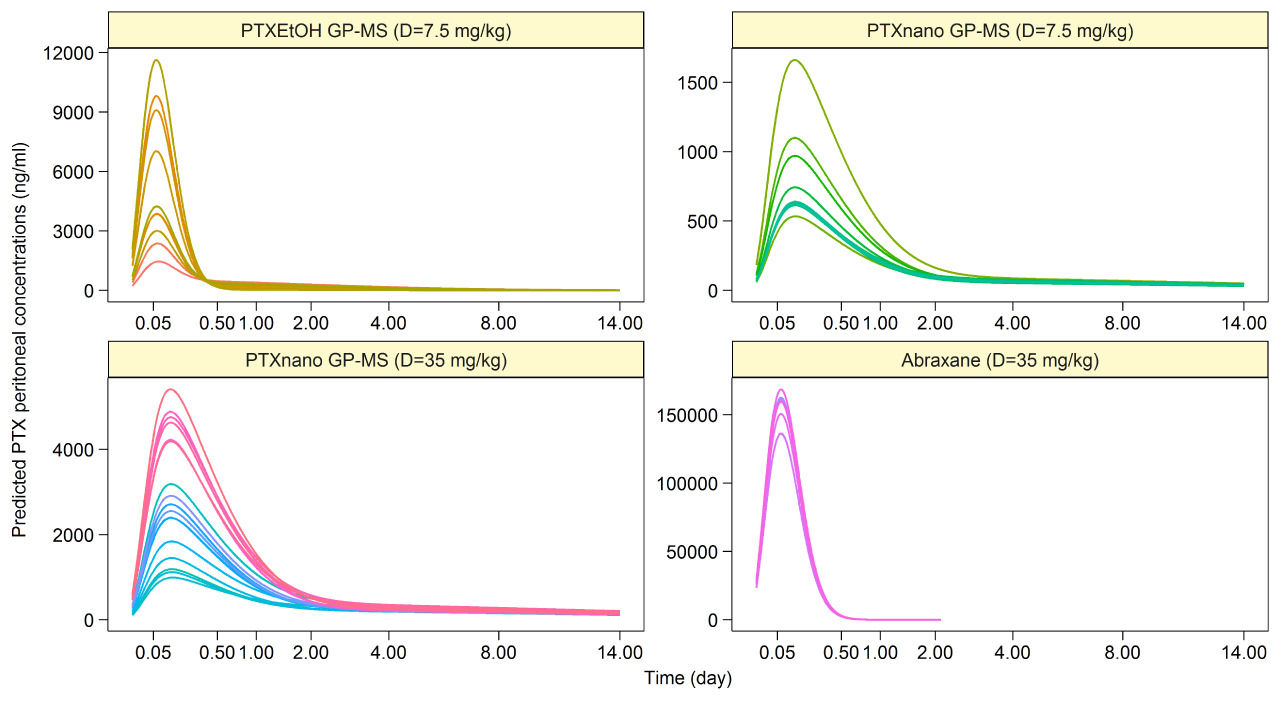


**Suppl. Fig. 3.** Individual peritoneal PTX concentration (ng/ml) as a function of time after IP administration of different types of PTX-GP-MS (PTX_EtOH_-GP-MS at a dose of 7.5 mg PTX/kg, PTX_nano_-GP-MS at doses of 7.5 and 35 mg PTX/kg), and nab-PTX at a dose of 35 mg PTX/kg


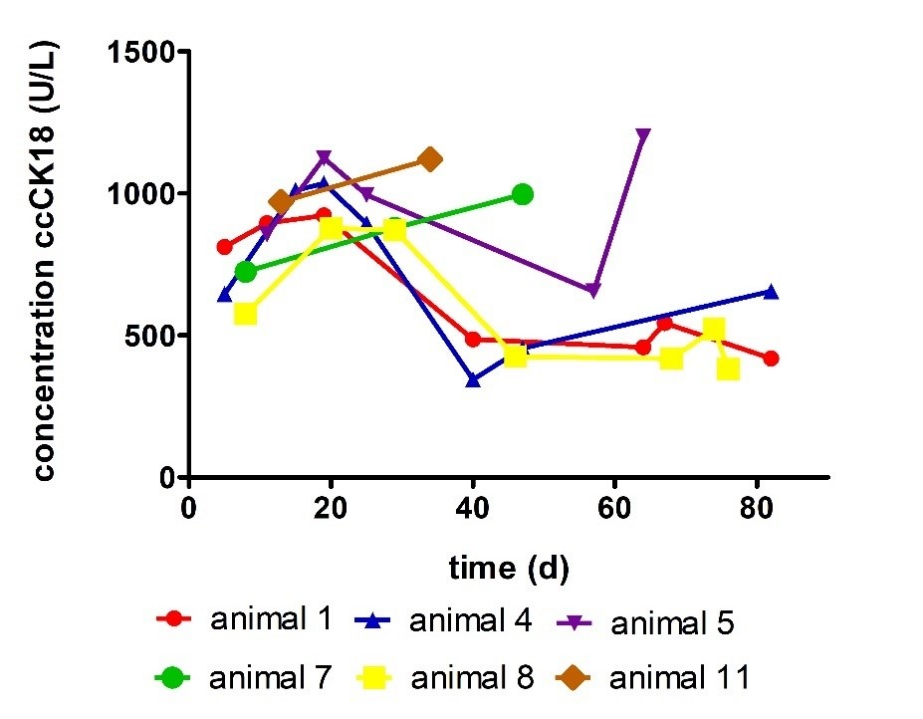


**Suppl. Fig. 4.** Representative profiles of released ccCK18 (U/L) as a function of time measured in plasma samples of mice receiving an IP PTX_EtOH_-GP-MS treatment in a human microscopic peritoneal carcinomatosis mouse model
